# Microwave-assisted synthesis of *N,N*-bis(phosphinoylmethyl)amines and *N,N,N*-tris(phosphinoylmethyl)amines bearing different substituents on the phosphorus atoms

**DOI:** 10.3762/bjoc.15.40

**Published:** 2019-02-15

**Authors:** Erika Bálint, Anna Tripolszky, László Hegedűs, György Keglevich

**Affiliations:** 1 Department of Organic Chemistry and Technology, Budapest University of Technology and Economics, 1521 Budapest, Hungary

**Keywords:** (aminomethyl)phosphine oxides, Kabachnik–Fields reaction, ligand, microwave, *N,N*-bis(phosphinoylmethyl)amines, *N,N,N*-tris(phosphinoylmethyl)amines

## Abstract

A family of *N*,*N*-bis(phosphinoylmethyl)amines bearing different substituents on the phosphorus atoms was synthesized by the microwave-assisted and catalyst-free Kabachnik–Fields reaction of (aminomethyl)phosphine oxides with paraformaldehyde and diphenylphosphine oxide. The three-component condensation of *N,N*-bis(phosphinoylmethyl)amine, paraformaldehyde and a secondary phosphine oxide affording *N,N,N*-tris(phosphinoylmethyl)amine derivatives was also elaborated. This method is a novel approach for the synthesis of the target products.

## Introduction

α-Aminophosphine oxides are of considerable importance as potential precursors of α-aminophosphine ligands [1]. α-Aminophosphines play an important role in the synthesis of P(III)-transition metal complexes [2], which are often applied catalysts in homogeneous catalytic reactions [2–4]. In addition, a few Pt, Ru and Au complexes incorporating phosphine ligands show significant anticancer activity [5–6].

One of the most common synthetic routes to α-aminophosphine oxides is the Kabachnik–Fields (phospha-Mannich) reaction, where an amine, an oxo compound (aldehyde or ketone) and a secondary phosphine oxide react in a condensation reaction [1]. However, only a few papers deal with the synthesis of α-aminophosphine oxides. (Phenylaminomethyl)dibenzylphosphine oxide was prepared by the three-component reaction of aniline, paraformaldehyde and dibenzylphosphine oxide [7], as well as by the reaction of (hydroxymethyl)dibenzylphosphine oxide and aniline [8]. The condensation of butylamine, paraformaldehyde and di(*p*-tolyl)phosphine oxide to afford (butylaminomethyl)di(*p*-tolyl)phosphine oxide was also described [9]. A microwave (MW)-assisted, catalyst-free method was elaborated by us for the synthesis of several (aminomethyl)phosphine oxides [10–11].

**Scheme 1:**
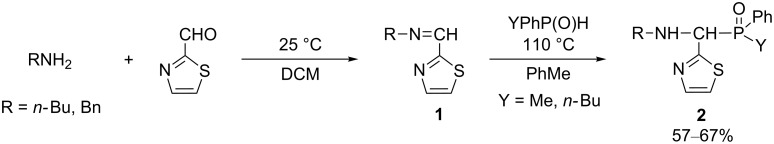
Synthesis of chiral thiazole-substituted aminophosphine oxides.

As regards α-aminophosphine oxides with different P-substituents, only two different types were reported. Olszewski and co-workers synthesized chiral thiazole-substituted aminophosphine oxides **2** through the Pudovik reaction of alkylphenylphosphine oxides and the corresponding aldimine derivatives of thiazole **1** (Scheme 1) [12].

**Scheme 2:**
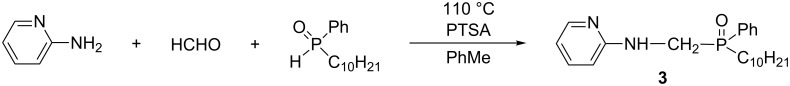
Synthesis of a P-chiral aminophosphine oxide containing a 2-pyridyl moiety.

Cherkasov and his group applied the Kabachnik–Fields reaction to synthesize a P-chiral aminophosphine oxide with a 2-pyridyl substituent **3** (Scheme 2) [13].

Bis(aminophosphine oxide) derivatives were also prepared by the double Kabachnik–Fields reaction using primary amines [11,14–15], amino acids [16–17] or aminoethanol [14] as the amine component.

**Scheme 3:**
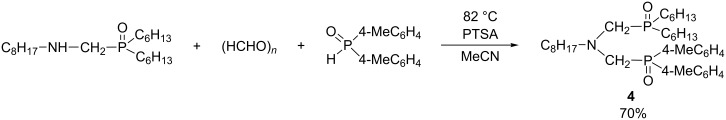
Condensation of (octylaminomethyl)dihexylphosphine oxide with paraformaldehyde and di(*p*-tolyl)phosphine oxide.

To the best of our knowledge, only one example can be found for a bis(α-aminophosphine oxide) containing different P-functions that was prepared by the condensation of (octylaminomethyl)dihexylphosphine oxide, paraformaldehyde and di(*p*-tolyl)phosphine oxide in the presence of *p*-toluenesulfonic acid in boiling acetonitrile (Scheme 3) [12].

Furthermore, tris(α-aminophosphine oxide) derivatives have not been described in the literature up to now. In this paper, we report the efficient, catalyst-free and MW-assisted synthesis of *N,N*-bis(phosphinoylmethyl)amine and *N,N,N*-tris(phosphinoylmethyl)amine derivatives bearing different substituents on the phosphorus atoms.

## Results and Discussion

### Synthesis of *N,N*-bis(phosphinoylmethyl)alkylamines containing different substituents on the phosphorus atoms

**Scheme 4:**
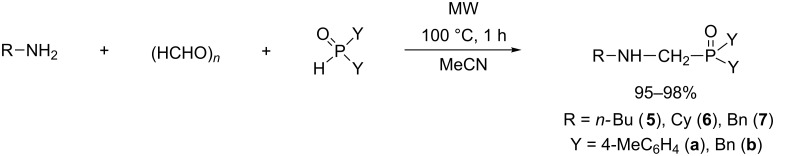
Synthesis of (aminomethyl)phosphine oxides **5**–**7**.

First, the (aminomethyl)phosphine oxide starting materials **5**–**7** were synthesized following our previous protocol [11]. Thus, the MW-assisted Kabachnik–Fields reaction of primary amines (butyl-, cyclohexyl- or benzylamine), paraformaldehyde and di(*p*-tolyl)- or dibenzylphosphine oxide was carried out in acetonitrile at 100 °C for 1 h affording the products with excellent yields (Scheme 4).

**Scheme 5:**
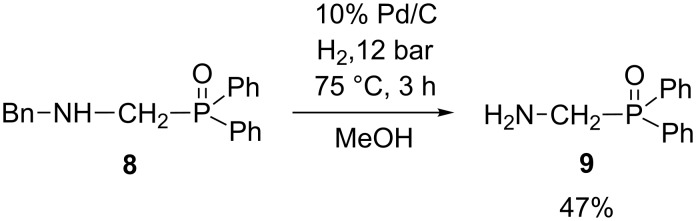
Synthesis of (aminomethyl)diphenylphosphine oxide (**9**).

Then, (aminomethyl)diphenylphosphine oxide (**9**) was prepared through debenzylation of (benzylaminomethyl)diphenylphosphine oxide (**8**, Scheme 5). The reduction was carried out in the presence of a 10% palladium on carbon catalyst (Selcat Q), in methanol, at 75 °C for 3 h, and the (aminomethyl)diphenylphosphine oxide (**9**) was obtained in a yield of 47% after column chromatography.

**Scheme 6:**
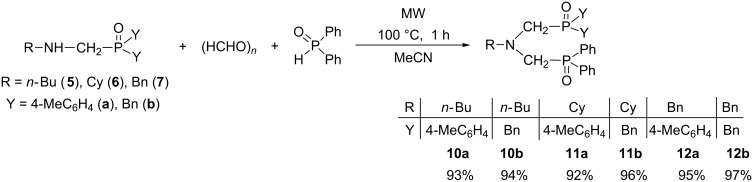
Synthesis of *N,N-*bis(phosphinoylmethyl)amines **10a**,**b**, **11a**,**b** and **12a**,**b** bearing different substituents at the phosphorus atoms (Y_2_P=O).

In the next step, (aminomethyl)phosphine oxides **5**–**7** were converted to bis(phosphinoylmethyl)amine derivatives bearing different substituents at the phosphorous atoms (Y_2_P=O) by reacting them with one equivalent of paraformaldehyde and diphenylphosphine oxide under MW conditions (Scheme 6). The three-component condensations were performed in the absence of any catalyst in acetonitrile as the solvent to overcome the heterogeneity of the reaction mixture. After an irradiation of 1 h at 100 °C, the mixed *N,N*-bis(phosphinoylmethyl)amines **10a**,**b**, **11a**,**b** and **12a**,**b** were obtained in yields of 92–97% and their structures were confirmed by ^31^P, ^13^C and ^1^H NMR, as well as HRMS measurements. Due to the two differently substituted phosphorous nuclei in the molecules, two signals were observed in the ^31^P NMR spectra.

**Scheme 7:**
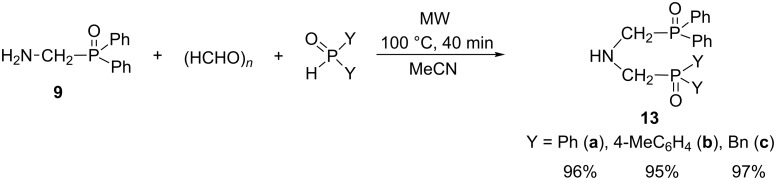
Synthesis of *N,N*-bis(phosphinoylmethyl)amines **13a**–**c**.

The valuable intermediate **9** was then utilized in the synthesis of *N,N*-bis(phosphinoylmethyl)amines **13a**–**c** (Scheme 7). The condensation of (aminomethyl)diphenylphosphine oxide (**9**), paraformaldehyde and various secondary phosphine oxides, such as diphenyl, di(*p*-tolyl) or dibenzylphosphine oxide, at 100 °C for 40 min led to the corresponding *N,N*-bis(phosphinoylmethyl)amines containing identical (**13a**) or different substituents on the phosphorus atoms (**13b** and **13c**) in excellent yields (95–97%).

### Synthesis of *N,N,N*-tris(phosphinoylmethyl)amines

**Scheme 8:**
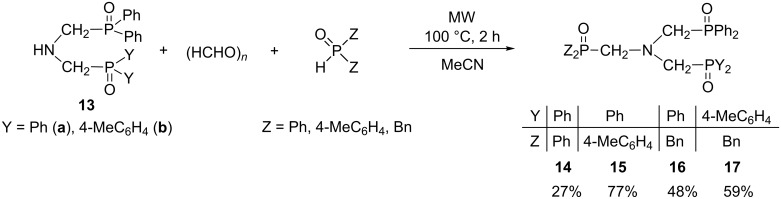
Synthesis of *N,N,N*-tris(phosphinoylmethyl)amines **14–17**.

Finally, *N,N*-bis(phosphinoylmethyl)amines **13a** and **13b** were reacted further with paraformaldehyde and a secondary phosphine oxide (diphenyl-, di(*p*-tolyl)- or dibenzylphosphine oxide) to afford the *N,N,N*-tris(phosphinoylmethyl)amine derivatives bearing identical (**14**) and different Y_2_P=O groups (**15**–**17**) (Scheme 8). The condensations were performed as mentioned above. The introduction of a third phosphinoylmethyl moiety into the bis-derivatives containing an NH unit (**13a** and **13b**) required a longer reaction time (2 h) at 100 °C. In these cases, the conversion was 70–95%, and the corresponding *N,N,N*-tris(phosphinoylmethyl)amine derivatives **14**–**17** were isolated in yields of 27–77%. However, applying a higher temperature and/or longer reaction time, lead to decomposition.

## Conclusion

In summary, we have developed an efficient, catalyst-free and MW-assisted method for the synthesis of *N,N*-bis(phosphinoylmethyl)amines and *N,N,N*-tris(phosphinoylmethyl)amines bearing different substituents on the phosphorus atoms by the Kabachnik–Fields reaction. This method is a novel approach for the synthesis of the target products. In all, thirteen new derivatives were isolated in high yields and fully characterized.

## Supporting Information

File 1 Experimental procedures, characterization data, details of the NMR structural determination of all products and copies of ^31^P, ^1^H, and ^13^C NMR spectra for all compounds synthesized.
