# Supplementary material for: Microwave-assisted synthesis of N,N-bis(phosphinoylmethyl)amines and N,N,N-tris(phosphinoylmethyl)amines bearing different substituents on the phosphorus atoms
Source: Beilstein J Org Chem. 2019 Feb 15;15:469–73. doi: 10.3762/bjoc.15.40 (PMC6404400; doi:10.3762/bjoc.15.40)
Supplement: File 1 — Experimental procedures, characterization data, details of the NMR structural determination of all products and copies of 31P, 1H, and 13C NMR spectra for all compounds synthesized. [file Beilstein_J_Org_Chem-15-469-s001.pdf]

BEILSTEIN JOURNAL OF ORGANIC CHEMISTRY

Supporting Information

for

Microwave-assisted synthesis of *N,N*-bis(phosphinoylmethyl)amines and *N,N,N*-tris(phosphinoylmethyl)amines bearing different substituents on the phosphorus atoms

Erika Bálint, Anna Tripolszky, László Hegedűs and György Keglevich

Beilstein J. Org. Chem. **2019,** *15,* 469–473. doi:10.3762/bjoc.15.40

Experimental procedures, characterization data, details of the NMR structural determination of all products and copies of ^{31}P, ^{1}H, and ^{13}C NMR spectra for all compounds synthesized

License and Terms: This is a supporting information file under the terms of the Creative Commons Attribution License (http://creativecommons.org/licenses/by/4.0). Please note that the reuse, redistribution and reproduction in particular requires that the authors and source are credited.
The license is subject to the *Beilstein Journal of Organic Chemistry* terms and conditions: (https://www.beilstein-journals.org/bjoc)

Table of contents

General information S2

General procedure for the synthesis of (aminomethyl)dibenzyl-, (aminomethyl)di(*p*-tolyl)- or (aminomethyl)diphenylphosphine oxides S2

The hydrogenation of (benzylaminomethyl)diphenylphosphine oxide.. S4

General procedure for the synthesis of *N*,*N*-bis(phosphinoylmethyl)alkylamines bearing different $Y_2P=O$ groups.. S4

General procedure for the synthesis of *N*,*N*-bis(phosphinoylmethyl)amines containing different $Y_2P=O$ groups.. S8

General procedure for the synthesis of *N*,*N*,*N* tris(phosphinoylmethyl)amines........................ S9

^{31}P NMR, ^{1}H NMR and ^{13}C NMR spectra.. S12

References.. S32

General information

The ^{31}P, ^{13}C, ^{1}H NMR spectra were recorded in $CDCl_3$ solution on a Bruker AV-300 or DRX-500 spectrometer operating at 121.5, 75.5 and 300 or 202.4, 125.7 and 500 MHz, respectively. Chemical shifts are reported downfield relative to 85% H_3PO_4 and TMS. The coupling constants are given in Hz. Mass spectrometric measurements were performed using a Q-TOF Premier mass spectrometer in positive electrospray mode and a Shimadzu LCMS-ITTOF mass spectrometer. The reactions were carried out in a 300 W CEM Discover microwave reactor (CEM Microwave Technology Ltd., Buckingham, UK) equipped with a pressure controller applying 30–80 W under isothermal conditions.

General procedure for the synthesis of (aminomethyl)dibenzyl-, (aminomethyl)di(*p*-tolyl)- or (aminomethyl)diphenylphosphine oxides

A mixture of 1.0 mmol amine (0.10 mL of butylamine, 0.12 mL of cyclohexylamine or 0.11 mL benzylamine), 1.0 mmol (0.03 g) of paraformaldehyde, and 1.0 mmol of the secondary phosphine oxide (0.24 g of di(*p*-tolyl)phosphine oxide, 0.24 g of dibenzylphosphine oxide or 0.20 g of diphenylphosphine oxide) and 1.5 mL of acetonitrile was heated at 100 °C in a closed vial in a CEM Discover microwave reactor equipped with a pressure controller for 1 h. Acetonitrile and the water formed during reaction were removed in vacuum. The crude product so obtained was passed through a 1 cm silica gel layer using ethyl acetate. After evaporating the solvent, the products **5a**,**b**, **6a**,**b**, **7a**,**b** and **8** were obtained as crystals or oils. The following products were thus prepared:

(Butylaminomethyl)di(*p*-tolyl)phosphine oxide (**5a**)

Yield: 97% (0.31 g) of compound **5a** as pale yellow oil; ^{31}P NMR ($CDCl_3$) δ 42.1; δ[S1] ($CDCl_3$) 42.0; $[M+H]^{+}_{found}$ = 316.1828, $C_{19}H_{27}NOP$ requires 316.1825.

(Butylaminomethyl)dibenzylphosphine oxide (**5b**)

Yield: 98% (0.31 g) of compound **5b** as pale yellow crystals; ^{31}P NMR ($CDCl_3$) δ 44.5; δ[S1] ($CDCl_3$) 44.6; $[M+H]^+_{found}$ = 316.1832, $C_{19}H_{27}NOP$ requires 316.1825.

(Cyclohexylaminomethyl)di(*p*-tolyl)phosphine oxide (**6a**)

Yield: 98% (0.33 g) of compound **6a** as pale yellow oil; ^{31}P NMR ($CDCl_3$) δ 29.5; δ[S1] ($CDCl_3$) 29.6; $[M+H]^+_{found}$ = 342.1987, $C_{21}H_{29}NOP$ requires 342.1981.

(Cyclohexylaminomethyl)dibenzylphosphine oxide (**6b**)

Yield: 96% (0.33 g) of compound **6b** as white crystals; ^{31}P NMR ($CDCl_3$) δ 44.2; δ[S1] ($CDCl_3$) 44.4; $[M+H]^+_{found}$ = 342.1989, $C_{21}H_{29}NOP$ requires 342.1981.

(Benzylaminomethyl)di(*p*-tolyl)phosphine oxide (**7a**)

Yield: 94% (0.33 g) of compound **7a** as pale yellow crystals; ^{31}P NMR ($CDCl_3$) δ 30.0; δ[S1] ($CDCl_3$) 29.9; $[M+H]^+_{found}$ = 350.1669, $C_{22}H_{25}NOP$ requires 350.1668.

(Benzylaminomethyl)dibenzylphosphine oxide (**7b**)

Yield: 97% (0.34 g) of compound **7b** as white crystals; ^{31}P NMR ($CDCl_3$) δ 43.6; δ[S1] ($CDCl_3$) 43.8; $[M+H]^+_{found}$ = 350.1677, $C_{22}H_{25}NOP$ requires 350.1668.

(Benzylaminomethyl)diphenylphosphine oxide (**8**)

Yield: 96% (0.32 g) of compound **8** as white crystals; ^{31}P NMR ($CDCl_3$) δ 30.0; δ[S2] ($CDCl_3$) 29.7; $[M+H]^+_{found}$ = 322.1316, $C_{20}H_{21}NOP$ requires 322.1361.

Hydrogenation of (benzylaminomethyl)diphenylphosphine oxide

To 1.6 g (5.00 mmol) of (benzylaminomethyl)diphenylphosphine oxide (**8**) in 100 mL of methanol was added 0.50 g of 10% palladium on carbon (Selcat Q) and the suspension was then hydrogenated in a stainless steel autoclave at 12 bar and 75 °C for 3 h. The mixture was filtered, and the catalyst was washed with methanol. After evaporating the solvent and column chromatography 0.54 g (47%) of **9** was obtained as a white crystal.

(Aminomethyl)diphenylphosphine oxide (**9**)

Yield: 47% (0.54 g) of compound **9** as white crystals; ^{31}P NMR ($CDCl_3$) δ 30.8; δ[S3] ($CDCl_3$) 30.4; $[M+H]^{+}_{found}$ = 232.0891, $C_{13}H_{15}NOP$ requires 232.0891.

General procedure for the synthesis of *N,N*-bis(phosphinoylmethyl)alkyl amines bearing different $Y_2P=O$ groups

A mixture of 0.50 mmol (aminomethyl)phosphine oxides [(butylaminomethyl)di(*p*-tolyl)phosphine oxide: 0.16 g, (cyclohexylaminomethyl)di(*p*-tolyl)phosphine oxide: 0.17 g, (benzylaminomethyl)di(*p*-tolyl)phosphine oxide: 0.17 g, (butylaminomethyl)dibenzylphosphine oxide: 0.16 g, (cyclohexylaminomethyl)dibenzylphosphine oxide: 0.17 g, (benzylaminomethyl)dibenzylphosphine oxide: 0.17 g], 0.015 g (0.50 mmol) of paraformaldehyde, and 0.10 g (0.50 mmol) of diphenylphosphine oxide and 1.5 mL of acetonitrile was heated at 100 °C in a closed vial in a CEM Discover microwave reactor equipped with a pressure controller for 1 h. Acetonitrile and the water formed during reaction were removed under vacuum. The crude product so obtained was passed through a 1 cm silica gel layer using ethyl acetate. After evaporating the solvent, the products **10a**,**b**, **11a**,**b** and **12a**,**b** were obtained as oils. The following products were thus prepared:

N,N-(Di-*p*-tolylphosphinoylmethyl)(diphenylphosphinoylmethyl)butylamine (**10a**)

Yield: 93% (0.25 g) of compound **10a** as colorless oil. ^{31}P NMR ($CDCl_3$) δ 29.3, 29.5; ^{13}C NMR ($CDCl_3$) δ 14.0 ($\mathit{C}H_3CH_2$), 20.0 ($CH_3\mathit{C}H_2$), 21.5 ($C_4\mathit{C}H_3$), 28.6 ($CH_3CH_2\mathit{C}H_2$), 55.9 (dd, $^1J_{CP}$ = 85.3, $^3J_{CP}$ = 1.3, N$\mathit{C}H_2PPh_2$), 56.0 (dd, $^1J_{CP}$ = 85.2, $^3J_{CP}$ = 2.7, N$\mathit{C}H_2$P(*p*-tolyl)$_2$), 58.4 (t, $^3J_{CP}$ = 7.0, $CH_3(CH_2)_2\mathit{C}H_2N$), 128.3 (d, $^2J_{CP}$ = 11.7, $C_{2'}$), 129.10 (d, $^2J_{CP}$ = 11.9, C_2), 129.13 (d, $^1J_{CP}$ = 100.1, C_1), 131.17 (d, $^3J_{CP}$ = 9.2, $C_{3'}$), 131.21 (d, $^3J_{CP}$ = 8.6, C_3), 131.5 (d, J_{CP} = 2.6, $C_{4'}$), 132.3 (d, $^1J_{CP}$ = 97.5, $C_{1'}$), 141.9 (d, J_{CP} = 2.8, C_4); ^{1}H NMR ($CDCl_3$) δ 0.73 (t, 3H, J_{HH} = 7.3, $C\mathit{H}_3CH_2$), 0.95–1.06 (m, 2H, $CH_3C\mathit{H}_2CH_2$), 1.20–1.30 (m, 2H, $CH_3CH_2C\mathit{H}_2$), 2.35 (s, 6H, $C_4C\mathit{H}_3$), 2.95 (t, 2H, $^3J_{HH}$ = 7.1, $CH_3CH_2CH_2C\mathit{H}_2N$), 3.63 (d, 2H, $^1J_{HP}$ = 5.9, NCH_2P(*p*-tolyl)$_2$), 3.73 (d, 2H, $^1J_{HP}$ = 6.0, NCH_2PPh_2), 7.12–7.19 (m, 4H, C_3H), 7.22–7.39 (m, 4H, $C_{3'}H$), 7.42–7.47 (m, 2H, $C_{4'}H$), 7.58–7.65 (m, 4H, C_2H), 7.72–7.80 (m, 4H, $C_{2'}H$); $[M+H]^+_{found}$ = 530.2226, $C_{32}H_{38}NO_2P_2$ requires 530.2378.

N,N-(Dibenzylphosphinoylmethyl)(diphenylphosphinoylmethyl)butylamine (**10b**)

Yield: 94% (0.25 g) of compound **10b** as colorless oil. ^{31}P NMR ($CDCl_3$) δ 29.3, 41.8; ^{13}C NMR ($CDCl_3$) δ 14.0 ($\mathit{C}H_3CH_2$), 20.1 ($CH_3\mathit{C}H_2$), 28.4 ($CH_3CH_2\mathit{C}H_2$), 34.9 (d, $^1J_{CP}$ = 60.4, P(O)CH_2), 53.6 (dd, $^1J_{CP}$ = 81.5, $^3J_{CP}$ = 9.8, NCH_2PBn_2), 55.1 (dd, $^1J_{CP}$ = 86.5, $^3J_{CP}$ = 6.0, NCH_2PPh_2), 58.5 (dd, $^3J_{CP}$ = 7.8, 5.8, $CH_3(CH_2)_2\mathit{C}H_2N$), 126.7 (d, J_{CP} = 2.7, C_4), 128.5 (d, $^2J_{CP}$ = 11.6, $C_{2'}$), 128.6 (d, J_{CP} = 2.0, C_3), 129.8 (d, $^3J_{CP}$ = 5.2, C_2), 131.3 (d, $^3J_{CP}$ = 7.3, $C_{3'}$), 131.8 (d, $^3J_{CP}$ = 2.7, $C_{4'}$), 131.9 (d, $^2J_{CP}$ = 7.1, $C_{1,}$) 132.1 (d, $^1J_{CP}$ = 97.6, $C_{1'}$); ^{1}H NMR ($CDCl_3$) δ 0.83 (t, J_{HH} = 7.2, 3H, CH_3), 1.06–1.36 (m, 4H, $CH_3C\mathit{H}_2C\mathit{H}_2$), 2.72–3.08 (m, 8H, $CH_3CH_2CH_2C\mathit{H}_2N$, P(O)$CH_2Ph$, NCH_2PPh_2,) 3.66 (d, $^3J_{HP}$ = 6.0, 2H, NCH_2PBn_2),

7.11–7.33 (m, 10H, C_2H, C_3H, C_4H), 7.40–7.52 (m, 6H, $C_{3'}H$, $C_{4'}H$), 7.82–7.95 (m, 4H, $C_{2'}H$); $[M+H]^+_{found}$ = 530.2167, $C_{32}H_{38}NO_2P_2$ requires 530.2378.

N,N-(Di-*p*-tolylphosphinoylmethyl)(diphenylphosphinoylmethyl)cyclohexyl-amine (**11a**)

Yield: 92% (0.26 g) of compound **11a** as pale yellow oil. ^{31}P NMR ($CDCl_3$) δ 29.9, 30.3; ^{13}C NMR ($CDCl_3$) δ 21.6 (C_4CH_3), 25.7 (C_3), 26.2 (C_4), 27.8 (C_2), 51.7 (dd, $^1J_{CP}$ = 87.2, $^3J_{CP}$ = 8.6, NCH_2PPh_2), 51.9 (dd, $^1J_{CP}$ = 87.2, $^3J_{CP}$ = 7.4, NCH_2P(*p*-tolyl)$_2$), 61.8 (t, $^3J_{CP}$ = 5.9, C_1), 128.3 (d, $^2J_{CP}$ = 11.6, $C_{2''}$), 129.06 (d, $^2J_{CP}$ = 11.9, $C_{2'}$), 129.07 (d, $^1J_{CP}$ = 100.0, $C_{1'}$), 131.2 (d, $^3J_{CP}$ = 9.3, $C_{3''}$), 131.3 (d, $^3J_{CP}$ = 9.0, $C_{3'}$), 131.4 (d, J_{CP} = 2.6, $C_{4''}$), 132.2 (d, $^1J_{CP}$ = 97.6, $C_{1''}$), 141.8 (d, J_{CP} = 2.7, $C_{4'}$); ^1H NMR ($CDCl_3$) δ 0.92–1.02 (m, 2H, $C_2H_{ax.}$), 1.22–1.34 (m, 3H, $C_3H_{ax.}$, $C_4H_{ax.}$), 1.54–1.71 (m, 5H, $C_2H_{eq.}$, $C_3H_{eq.}$, $C_4H_{eq.}$), 2.35 (s, 6H, C_4CH_3), 3.35–3.45 (m, 1H, C_1H), 3.67 (d, $^1J_{HP}$ = 6.5, 2H, NCH_2P(*p*-tolyl)$_2$), 3.77 (d, $^1J_{HP}$ = 6.6, 2H, NCH_2PPh_2), 7.13–7.20 (m, 4H, $C_{3'}H$), 7.32–7.38 (m, 4H, $C_{3''}H$), 7.41–7.47 (m, 2H, $C_{4''}H$), 7.58–7.66 (m, 4H, $C_{2'}H$), 7.72–7.80 (m, 4H, $C_{2''}H$); $[M+H]^+_{found}$ = 556.2300, $C_{34}H_{40}NO_2P_2$ requires 556.2534.

N,N-(Dibenzylphosphinoylmethyl)(diphenylphosphinoylmethyl)cyclohexyl-amine (**11b**)

Yield: 96% (0.27 g) of compound **11b** as pale yellow oil. ^{31}P NMR ($CDCl_3$) δ 30.1, 41.7; ^{13}C NMR ($CDCl_3$) δ 25.6 (C_3), 26.1 (C_4), 27.9 (C_2), 34.8 (d, $^1J_{CP}$ = 60.6, $P(O)CH_2$), 49.5 (dd, $^1J_{CP}$ = 83.5, $^3J_{CP}$ = 9.7, NCH_2PBn_2), 51.8 (dd, $^1J_{CP}$ = 88.1, $^3J_{CP}$ = 6.2, NCH_2PPh_2), 61.8 (t, $^3J_{CP}$ = 5.3, C_1), 126.6 (d, J_{CP} = 2.6, $C_{4'}$), 128.5 (d, $^2J_{CP}$ = 11.7, $C_{2''}$), 128.6 (d, J_{CP} = 2.3, $C_{3'}$), 129.8 (d, $^3J_{CP}$ = 5.2, $C_{2'}$), 131.4 (d, $^3J_{CP}$ = 9.02 $C_{3''}$), 131.8 (d, J_{CP} = 2.6, $C_{4''}$), 132.1 (d, $^2J_{CP}$ = 7.0, $C_{1'}$), 131.9 (d, $^1J_{CP}$ = 107.2, $C_{1''}$); ^1H NMR ($CDCl_3$) δ 0.80–1.26 (m, 5H, $C_2H_{ax.}$, $C_4H_{ax.}$, $C_3H_{ax.}$), 1.51–1.75 (m, 5H, $C_2H_{eq.}$, $C_4H_{eq.}$, $C_3H_{eq.}$), 2.70–3.32 (m, 7H, C_1H, $P(O)CH_2Ph$, NCH_2PPh_2,), 3.69 (d, $^1J_{HP}$ = 6.1, 2H, NCH_2PBn_2), 7.12–7.31 (m, 10H, $C_{2'}H$,

$C_{3'}H$, $C_{4'}H$), 7.40–7.53 (m, 6H, $C_{3''}H$, $C_{4''}H$), 7.83–7.96 (m, 4H, $C_{2''}H$); $[M+H]^+_{found}$ = 566.2406 , $C_{34}H_{40}NO_2P_2$ requires 556.2534.

N,N-(Di-*p*-tolylphosphinoylmethyl)(diphenylphosphinoylmethyl)benzylamine (**12a**)

Yield: 95% (0.27 g) of compound **12a** as colorless oil. ^{31}P NMR ($CDCl_3$) δ 29.8, 30.1; ^{13}C NMR ($CDCl_3$) δ 21.6 (C_4CH_3), 55.1 (dd, $^1J_{CP}$ = 85.1, $^3J_{CP}$ = 7.0, NCH_2PPh_2), 55.2 (dd, $^1J_{CP}$ = 85.0, $^3J_{CP}$ = 8.1, NCH_2P(*p*-tolyl)$_2$), 63.2 (t, $^3J_{CP}$ = 7.5, NCH_2Ph), 127.2 (C_4), 128.1 (C_2), 128.4 (d, $^2J_{CP}$ = 11.7, $C_{2''}$), 129.0 (d, $^1J_{CP}$ = 94,9, $C_{1'}$), 129.1 (d, $^2J_{CP}$ = 12.0, $C_{2'}$), 129.9 (C_3), 131.1 (d, $^3J_{CP}$ = 9.1, $C_{3''}$), 131.2 (d, $^3J_{CP}$ = 9.2, $C_{3'}$), 131.5 (d, J_{CP} = 2.7, $C_{4''}$), 132.1 (d, $^1J_{CP}$ = 98.2, $C_{1''}$) 137.7 (C_1), 141.9 (d, J_{CP} = 2.8, $C_{4'}$); ^{1}H NMR ($CDCl_3$) δ 2.34 (s, 6H, C_4CH_3), 3.66 (d, 2H, $^1J_{HP}$ = 6.2, NCH_2P(*p*-tolyl)$_2$), 3.75 (d, 2H, $^1J_{HP}$ = 6.2, NCH_2PPh_2), 4.09 (s, 2H, CH_2N), 6.85 (d, 2H, J_{HH} = 7.3, C_2H), 6.85 (d, J_{HP} = 7.3, 2H, C_3H), 7.07–7.20 (m, 5H, $C_{3'}H$, C_4H), 7.25–7.35 (m, 6H, $C_{3''}H$, $C_{4''}H$), 7.42 (d, J_{HP} = 7.4, 2H, C_2H), 7.49 (dd, $^2J_{HP}$ = 11.1, J_{HP} = 8.1, 4H, $C_{2'}H$), 7.64 (dd, $^2J_{HP}$ = 11.0, J_{HP} = 7.8, 4H, $C_{2''}H$); $[M+H]^+_{found}$ = 564.2001, $C_{35}H_{36}NO_2P_2$ requires 564.2221.

N,N-(Dibenzylphosphinoylmethyl)(diphenylphosphinoylmethyl)benzylamine (**12b**)

Yield: 97% (0.27 g) of compound **12b** as colorless oil. ^{31}P NMR ($CDCl_3$) δ ^{31}P NMR ($CDCl_3$) δ 29.8, 41.2; ^{13}C NMR ($CDCl_3$) δ 34.9 (d, $^1J_{CP}$ = 60.6, P(O)CH_2), 52.7 (dd, $^1J_{CP}$ = 81.2., $^3J_{CP}$ = 9.9, NCH_2PBn_2), 54.8 (dd, $^1J_{CP}$ = 85.8., $^3J_{CP}$ = 5.7, NCH_2PPh_2), 61.2 (dd, $^3J_{CP}$ = 8.3, 6,0, $CH_3(CH_2)_2CH_2N$), 126.7 (d, J_{CP} = 2.8, $C_{4'}$), 127.5 (C_4), 128.3 (C_3), 128.5 (d, J_{CP} = 11.7, $C_{2''}$), 128.6 (d, J_{CP} = 2.4, $C_{3'}$), 129.7 (d, $^3J_{CP}$ = 5.2, $C_{2'}$), 130.0 (C_2), 131.3 (d, $^3J_{CP}$ = 9.1, $C_{3''}$), 131.80 (d, $^2J_{CP}$ = 6.2, $C_{1'}$), 131.82 (d, J_{CP} = 3.2, $C_{4''}$), 131.85 (d, $^1J_{CP}$ = 98.2, $C_{1''}$), 137.6 (C_1); ^{1}H NMR ($CDCl_3$) δ 2.66–2.89 (m, 4H, PCH_2Ph), 2.92 (d, 2H, $^1J_{HP}$ = 5,2, NCH_2PPh_2), 3.70 (d, 2H, $^1J_{HP}$ = 6,2, NCH_2PBn_2), 4.02 (s, 2H, NCH_2Ph), 7.01 (d, J_{HP} = 7.5, 4H, $C_{3'}H$), 7.05–

7.12 (m, 2H, C_3H), 7.16–7.30 (m, 9H, C_2H, $C_{2'}H$, C_4H, $C_{4'}H$), 7.39–7.51 (m, 6H, $C_{3''}H$, $C_{4''}H$), 7.81 (dd, $^2J_{HP}$ = 11.1, J_{HP} = 7.1, 4H, $C_{2''}H$); $[M+H]^+_{found}$ = 564.1888, $C_{35}H_{36}NO_2P_2$ requires 564.2221.

General procedure for the synthesis of *N,N*-bis(phosphinoylmethyl)amines containing different Y_2P=O groups

A mixture of 0.12 g (0.50 mmol) of (aminomethyl)diphenylphosphine oxide (**9**), 0.015 g (0.5 mmol) of paraformaldehyde, and 0.10 g (0.50 mmol) of 0.5 mmol of the secondary phosphine oxide (0.10 g of diphenylphosphine oxide, 0.12 g of di(*p*-tolyl)phosphine oxide or 0.12 g of dibenzylphosphine oxide) and 1.5 mL of acetonitrile was heated at 100 °C in a closed vial in a CEM Discover microwave reactor equipped with a pressure controller for 40 min. Acetonitrile and the water formed during the reaction were removed in vacuum. The crude product so obtained was passed through a 1 cm silica gel layer using ethyl acetate. After evaporating the solvent, the products **13a**–**c** were obtained as crystals or oils. The following products were thus prepared:

N,N-Bis(diphenylphosphinoylmethyl)amine (**13a**)

Yield: 96% (0.21 g) of compound **13a** as colorless oil. ^{31}P NMR ($CDCl_3$) δ 29.6; ^{13}C NMR ($CDCl_3$) δ 55.1 (dd, $^1J_{CP}$ = 81.5, $^3J_{CP}$ = 10.7, NCH_2PPh_2), 128.6 (d, $^2J_{CP}$ = 11.7, C_2), 131.2 (d, $^3J_{CP}$ = 9.4, C_3), 131.4 (d, $^1J_{CP}$ = 98.1, $C_{1,}$), 132.0 (d, $^3J_{CP}$ = 2.6, C_4); ^1H NMR ($CDCl_3$) δ 3.68 (d, $^1J_{HP}$ = 6.8, 4H, $NHCH_2P$), 5.29 (s, 1H, NH), 7.38–7.45 (m, 8H, C_3H), 7.48–7.54 (m, 4H, C_4H,), 7.67–7.74 (m, 8H, C_2H,); $[M+H]^+_{found}$ = 446.1427, $C_{26}H_{26}NO_2P_2$ requires 446.1438.

N,N-(Diphenylphosphinoylmethyl)(di-*p*-tolylphosphinoylmethyl)amine (**13b**)

Yield: 95% (0.22 g) of compound **13b** as colorless oil. ^{31}P NMR ($CDCl_3$) δ 29.4, 29.8; ^{13}C NMR ($CDCl_3$) δ 21.6 (C_4CH_3), 50.5 (dd, $^1J_{CP}$ = 81.5, $^3J_{CP}$ = 10.4, NCH_2PPh_2), 50.7 (dd,

$^{1}J_{CP}$ = 81.0, $^{3}J_{CP}$ = 10.9, NCH_2P(*p*-tolyl)$_2$), 128.2 (d, $^{1}J_{CP}$ = 100.4, $C_{1'}$), 128.5 (d, $^{2}J_{CP}$ = 11.6, C_2), 129.3 (d, $^{2}J_{CP}$ = 12.0, $C_{2'}$), 131.1 (d, $^{3}J_{CP}$ = 2.3, C_3), 131.2 (d, $^{3}J_{CP}$ = 1.9, $C_{3'}$), 131.4 (d, $^{1}J_{CP}$ = 98.2, C_1), 131.9 (d, J_{CP} = 2.7, C_4), 142.4 (d, J_{CP} = 2.8, $C_{4'}$); ^{1}H NMR ($CDCl_3$) δ 2.38 (s, 6H, C_4CH_3), 3.62 (d, 2H, $^{1}J_{HP}$ = 7.0, NCH_2P(*p*-tolyl)$_2$), 3.67 (d, 2H, $^{1}J_{HP}$ = 7.1, NCH_2PPH_2), 5.29 (s, 1H, NH), 7.17–7.21 (m, 4H, $C_{3'}H$), 7.36–7.46 (m, 4H, C_3H), 7.47–7.63 (m, 6H, $C_{2'}H$, C_4H), 7.65–7.76 (m, 4H, C_2H); $[M+H]^+_{found}$ = 474.1752, $C_{28}H_{30}NO_2P_2$ requires 474.1751.

N,N-(Diphenylphosphinoylmethyl)(dibenzylphosphinoylmethyl)amine (**13c**)

Yield: 97% (0.23 g) of compound **13c** as white crystals. Mp: 149–153 °C. ^{31}P NMR ($CDCl_3$) δ 29.5, 43.8; ^{13}C NMR ($CDCl_3$) δ 34.2 (d, $^{1}J_{CP}$ = 60.0, P(O)CH_2), 46.5 (dd, $^{1}J_{CP}$ = 79.2, $^{3}J_{CP}$ = 12.3, NCH_2PBn_2), 50.3 (dd, $^{1}J_{CP}$ = 80.7, $^{3}J_{CP}$ = 9.4, NCH_2PPh_2), 126.9 (d, J_{CP} = 2.9, $C_{4'}$), 128.6 (d, $^{2}J_{CP}$ = 11.7, C_2), 128.8 (d, J_{CP} = 2.4, $C_{3'}$), 129.6 (d, $^{3}J_{CP}$ = 5.1, $C_{2'}$), 131.1 (d, $^{3}J_{CP}$ = 9.3, C_3), 131.4 (d, $^{1}J_{CP}$ = 99.8, $C_{1,}$), 131.5 (d, $^{2}J_{CP}$ = 7.1, $C_{1'}$), 132.1 (d, J_{CP} = 2.8, C_4); ^{1}H NMR ($CDCl_3$) δ 2.89 (d, $^{1}J_{HP}$ = 6.6, 2H, NCH_2PPh_2), 2.94–3.13 (m, 4H, P(O)CH_2Ph) 3.58. (d, $^{1}J_{HP}$ = 7.0, 2H, NCH_2PBn_2), 5.29 (s, 1H, NH), 7.09–7.18 (m, 4H, $C_{3'}H$), 7.19–7.33 (m, 6H, $C_{2'}H$, $C_{4'}H$), 7.43–7.59 (m, 6H, C_3H, C_4H), 7.73–7.85 (m, 4H, C_2H); $[M+H]^+_{found}$ = 474.1743, $C_{28}H_{30}NO_2P_2$ requires 474.1751.

General procedure for the synthesis of *N,N,N*-tris(phosphinoylmethyl)amines

A mixture of 0.50 mmol of *N,N*-bis(phosphinoylmethyl)amines (0.22 g of *N,N*-bis(diphenylphosphinoylmethyl)amine or 0.24 g of *N,N*-(diphenylphosphinoylmethyl)(di-*p*-tolylphosphinoylmethyl)amine), 0.015 g (0.50 mmol) of paraformaldehyde, and 0.50 mmol of the secondary phosphine oxide (0.12 g of dibenzylphosphine oxide, 0.12 g of di(*p*-tolyl)phosphine oxide or 0.10 g of diphenylphosphine oxide) and 1.5 mL of acetonitrile was heated at 100 °C in a closed vial in a CEM Discover microwave reactor equipped with a pressure controller for 2 h. Acetonitrile and the water formed during the reaction were

removed in vacuum. The crude product so obtained was passed through a 1 cm silica gel layer using ethyl acetate. After column chromatography, the products **14**–**17** were obtained as crystals. The following products were thus prepared:

N,*N*,*N*-Tris(diphenylphosphinoylmethyl)amine (**14**)

Yield: 27% (0.09 g) of compound **14** as white crystals. Mp: 71–74 °C. ^{31}P NMR ($CDCl_3$) δ 30.3; ^{13}C NMR ($CDCl_3$) δ 58.1 (dt, $^1J_{CP}$ = 83.5, $^3J_{CP}$ = 7.6, NCH_2PPh_2), 128.3 (d, $^2J_{CP}$ = 11.9, C_2), 131.1 (d, $^3J_{CP}$ = 9.4, C_3), 131.54 (d, J_{CP} = 2.6, C_4), 131.54 (d, $^1J_{CP}$ = 99.7, C_1); ^{1}H NMR ($CDCl_3$) δ 4.21 (d, 6H, $^1J_{HP}$ = 6.6, NCH_2PPh_2), 7.19–7.29 (m, 12H, C_3H), 7.33–7.41 (m, 6H, C_4H,), 7.75 (dd, 12H, $^2J_{HP}$ = 11.1, J_{HP} = 7.8, C_2H,); $[M+H]^+_{found}$ = 660.1972, $C_{39}H_{37}NO_3P_3$ requires 660.1986.

N,*N*,*N*-[Bis(diphenylphosphinoylmethyl)(di-*p*-tolylphosphinoylmethyl)]amine (**15**)

Yield: 77% (0.26 g) of compound **15** as white crystals. Mp: 76–79 °C. ^{31}P NMR ($CDCl_3$) δ 28.0, 28.3; ^{13}C NMR ($CDCl_3$) δ 21.5 (C_4CH_3), 58.1 (dd, $^1J_{CP}$ = 84.0, $^3J_{CP}$ = 7.8, NCH_2PPh_2), 58.3 (dd, $^1J_{CP}$ = 83.8, $^3J_{CP}$ = 7.6, NCH_2P(*p*-tolyl)$_2$), 128.5 (d, $^1J_{CP}$ = 102.1, $C_{1'}$), 128.4 (d, $^2J_{CP}$ = 11.9, C_2), 129.1 (d, $^2J_{CP}$ = 12.2, $C_{2'}$), 131.1 (d, $^3J_{CP}$ = 9.8, $C_{3'}$), 131.2 (d, $^3J_{CP}$ = 9.4, C_3), 131.5 (d, J_{CP} = 2.7, C_4), 131.7 (d, $^1J_{CP}$ = 99.5, C_1), 141.8 (d, J_{CP} = 2.8, $C_{4'}$); ^{1}H NMR ($CDCl_3$) δ 2.30 (s, 6H, C_4CH_3), 4.14 (d, 2H, $^1J_{HP}$ = 6.3, NCH_2P(*p*-tolyl)$_2$), 4.19 (d, 4H, $^1J_{HP}$ = 6.4, NCH_2PPH_2), 7.03 (d, 4H, $^3J_{HP}$ = 6.0, $C_{3'}$H), 7.21–7.29 (m, 8H, C_3H), 7.38 (t, 4H, J_{HP} = 67.2, C_4H), 7.61 (dd, 4H, $^2J_{HP}$ = 11.2, J_{HP} = 8.0, $C_{2'}$H), 7.74 (dd, 8H, $^2J_{HP}$ = 11.2, J_{HP} = 7.60, C_2H); $[M+H]^+_{found}$ = 688.2281, $C_{41}H_{41}NO_2P_2$ requires 688.2299.

N,N,N-[Bis(diphenylphosphinoylmethyl)(dibenzylphosphinoylmethyl)]amine (**16**)

Yield: 48% (0.16 g) of compound **16** as white crystals. Mp: 72–75 °C. ^{31}P NMR ($CDCl_3$) δ 30.5, 40.8; ^{13}C NMR ($CDCl_3$) δ 33.8 (d, $^{1}J_{CP}$ = 61.6, P(O)CH_2), 55.6 (dd, $^{1}J_{CP}$ = 80.5, $^{3}J_{CP}$ = 7.1, NCH_2PBn_2), 56.6 (dd, $^{1}J_{CP}$ = 84.1, $^{3}J_{CP}$ = 7.6, NCH_2PPh_2), 125.7 (d, J_{CP} = 2.7, $C_{4'}$), 127.5 (d, $^{2}J_{CP}$ = 11.7, C_2), 127.6 (d, J_{CP} = 2.4, $C_{3'}$), 128.7 (d, $^{3}J_{CP}$ = 5.3, $C_{2'}$), 130.1 (d, $^{3}J_{CP}$ = 9.4, C_3), 130.3 (d, $^{1}J_{CP}$ = 99.4, $C_{1,}$), 130.7 (d, $^{2}J_{CP}$ = 7.8, $C_{1'}$), 130.9 (d, J_{CP} = 2.8, C_4); ^{1}H NMR ($CDCl_3$) δ 2.63 (d, $^{1}J_{HP}$ = 13.2, 4H, NCH_2PPh_2), 3.48 (dd, $^{1}J_{HP}$ = 59.9, $^{3}J_{HP}$ = 4.7, 4H, P(O)CH_2Ph) 3.96 (d, $^{1}J_{HP}$ = 6.5, 2H, NCH_2PBn_2), 7.03 (d, $^{3}J_{HP}$ = 7.1, 4H, $C_{3'}$H), 7.09–7.22 (m, 6H, $C_{2'}$H, $C_{4'}$H), 7.23–7.39 (m, 12H, C_3H, C_4H), 7.73–7.85 (m, 8H, C_2H); $[M+H]^{+}_{found}$ = 688.2276, $C_{41}H_{41}NO_3P_3$ requires 688.2299.

N,N,N-[(Diphenylphosphinoylmethyl)(di-*p*-tolylphosphinoylmethyl) (dibenzyl-phosphinoylmethyl)]amine (**17**)

Yield: 59% (0.21 g) of compound **17** as white crystals. Mp: 69–72 °C. ^{31}P NMR ($CDCl_3$) δ 30.2, 30.4, 40.52; ^{13}C NMR ($CDCl_3$) δ 21.5 (C_4CH_3), 34.8 (d, $^{1}J_{CP}$ = 61.4, P(O)CH_2Ph), 56.7 (dd, $^{1}J_{CP}$ = 78.8, $^{3}J_{CP}$ = 7.5, NCH_2PBn_2), 57.7 (dd, $^{1}J_{CP}$ = 83.8, $^{3}J_{CP}$ = 7.6, NCH_2PPh_2, 57.8 (dd, $^{1}J_{CP}$ = 85.7, $^{3}J_{CP}$ = 8.4, NCH_2P(*p*-tolyl)$_2$), 126.9 (d, J_{CP} = 2.9, $C_{4''}$) 128.3 (d, $^{1}J_{CP}$ = 101.8, $C_{1'}$), 128.5 (d, $^{2}J_{CP}$ = 11.8, C_2), 128.6 (d, J_{CP} = 1.9, $C_{3''}$), 129.3 (d, $^{2}J_{CP}$ = 12.1, $C_{2'}$), 129.7 (d, $^{3}J_{CP}$ = 5.2, $C_{2''}$), 131.16 (d, $^{3}J_{CP}$ = 9.2, $C_{3'}$), 131.23 (d, $^{3}J_{CP}$ = 8.5, C_3), 131.5 (d, $^{1}J_{CP}$ = 99.3, C_1), 131.7 (d, J_{CP} = 2.7, C_4), 131.8 (d, $^{2}J_{CP}$ = 9.7, $C_{1''}$) 142.1 (d, J_{CP} = 2.7, $C_{4'}$); ^{1}H NMR ($CDCl_3$) δ 2.31 (s, 6H, C_4CH_3), 2.73 (d, 4H, $^{1}J_{HP}$ = 13.2, PCH_2Ph), 3.47 (d, 2H, $^{1}J_{HP}$ = 6.4, NCH_2PBn_2), 3.99 (d, 2H, $^{1}J_{HP}$ = 6.4, NCH_2P(*p*-tolyl)$_2$), 4.03 (d, 2H, $^{1}J_{HP}$ = 6.2, NCH_2PPh_2), 7.12 (d, 4H, $^{3}J_{HP}$ = 7.3, C_3H), 7.14–7.17 (m, 4H, $C_{3''}$H), 7.24–7.30 (m, 6H, $C_{3'}$H, $C_{4'}$H), 7.35–7.41 (m, 4H, $C_{2'}$H), 7.42–7.46 (m, 2H, C_4H), 7.73 (dd, 4H, $^{2}J_{HP}$ = 11.2, J_{HP} = 8.0, $C_{2''}$H), 7.88 (dd, 8H, $^{2}J_{HP}$ = 11.4, J_{HP} = 7.17, C_2H); $[M+H]^{+}_{found}$ = 716.2584, $C_{43}H_{45}NO_3P_3$ requires 716.2612.

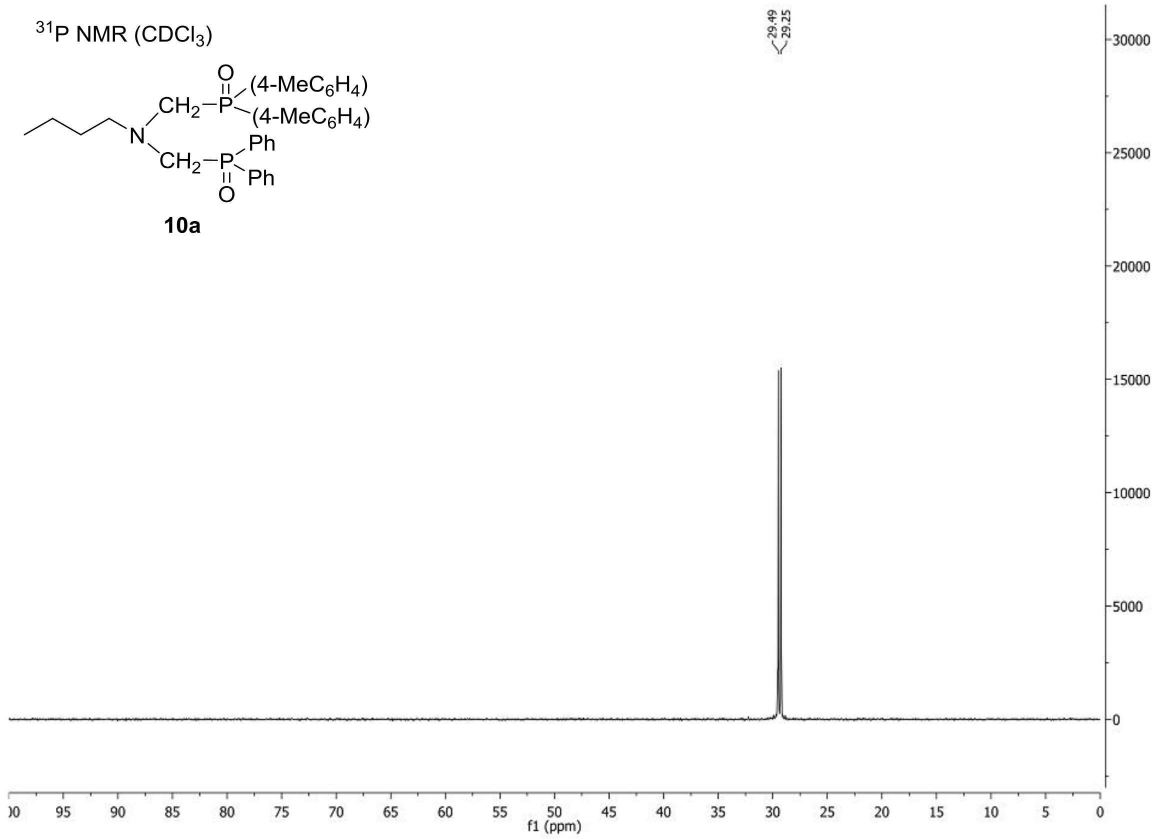

31P NMR (CDCl3)
(4-MeC6H4)
(4-MeC6H4)
Ph
Ph
10a
29.49
29.25
f1 (ppm)

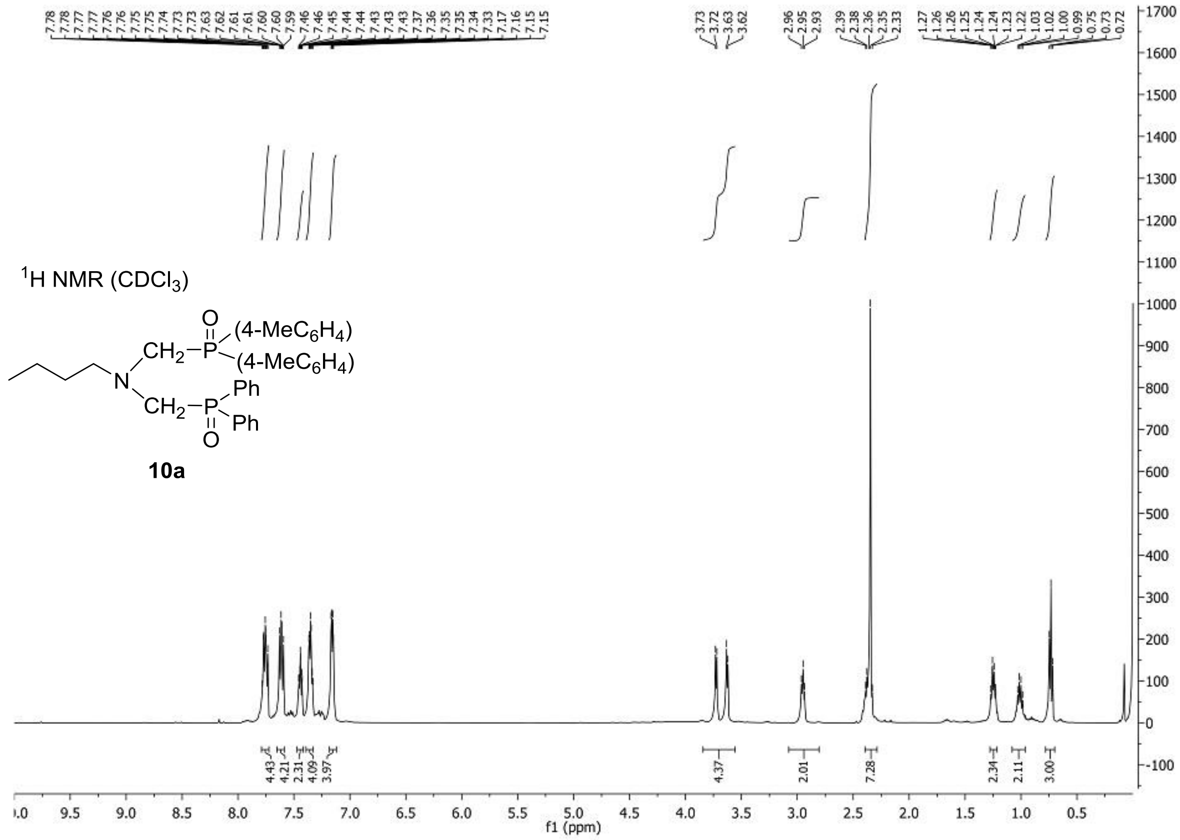

1H NMR (CDCl3)
(4-MeC6H4)
(4-MeC6H4)
Ph
Ph
10a
f1 (ppm)
4.43
4.21
2.31
4.09
3.97
4.37
2.01
7.28
2.34
2.11
3.00

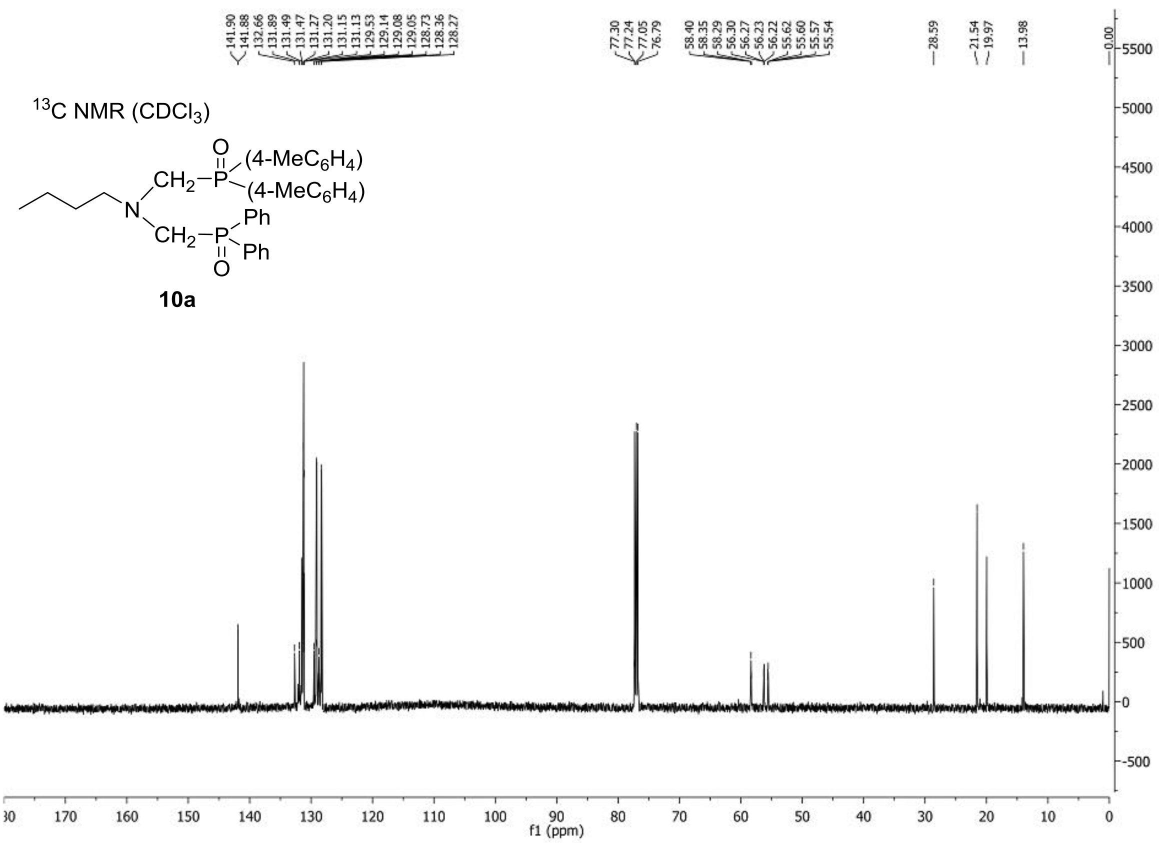

13C NMR (CDCl3)
(4-MeC6H4)
(4-MeC6H4)
Ph
Ph
10a
141.90
141.88
132.66
131.89
131.49
131.47
131.27
131.20
131.15
131.13
129.53
129.14
129.08
129.05
128.73
128.36
128.27
77.30
77.24
77.05
76.79
58.40
58.35
58.29
56.30
56.27
56.23
56.22
55.62
55.60
55.57
55.54
28.59
21.54
19.97
13.98
0.00
f1 (ppm)

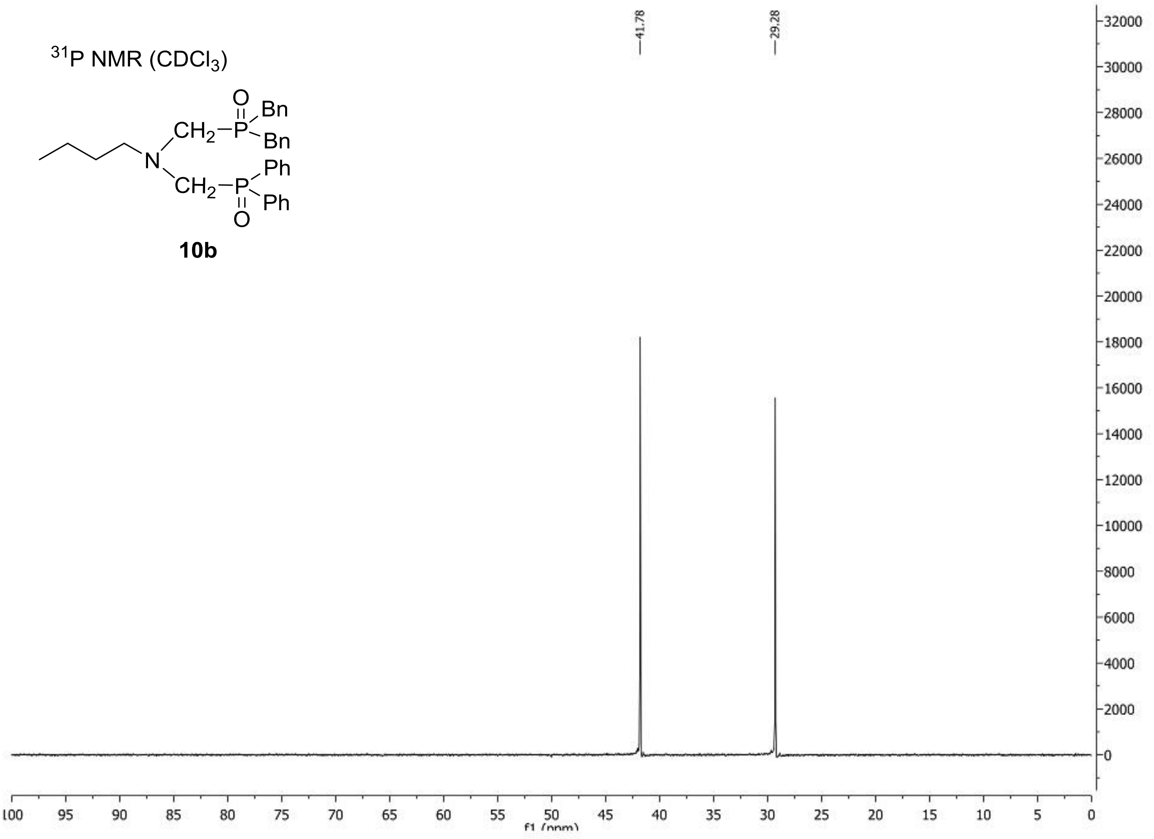

31P NMR (CDCl3)
Bn
Bn
Ph
Ph
10b
41.78
29.28
f1 (ppm)

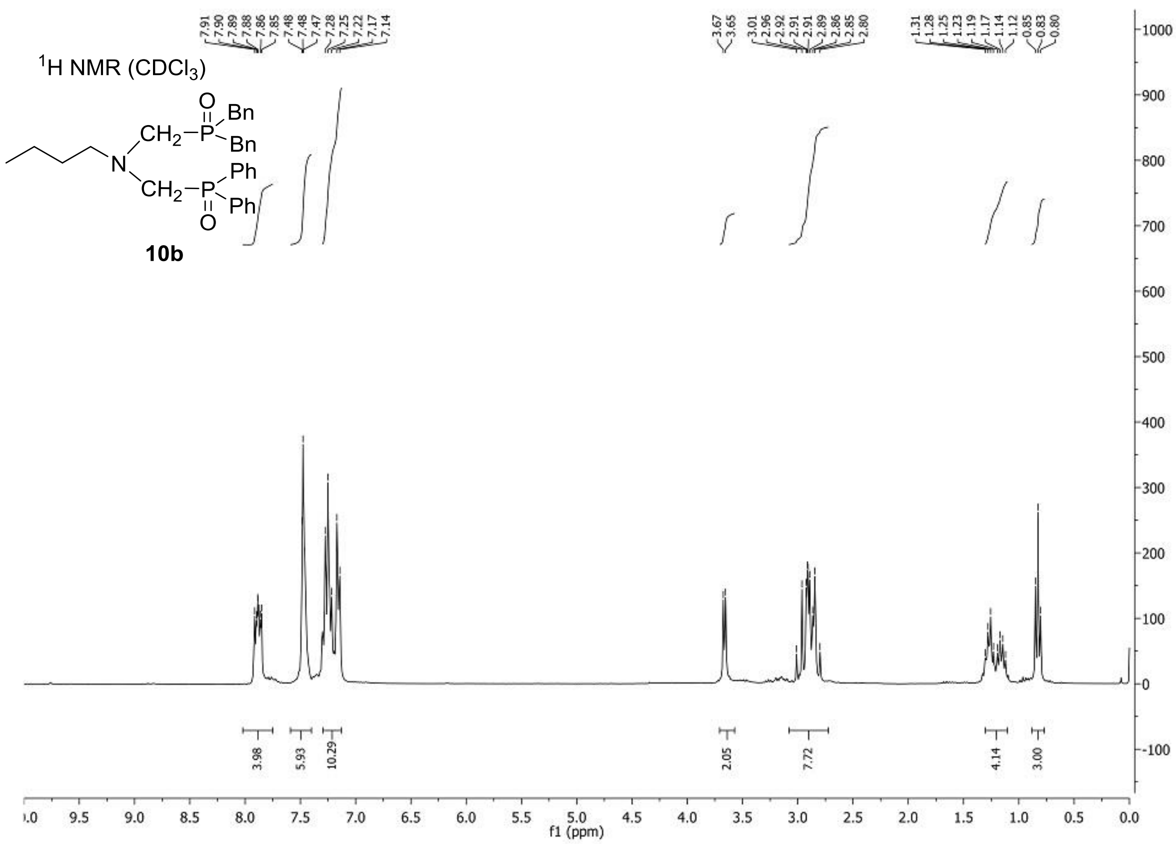
^{1}H NMR ($CDCl_3$)
10b
f1 (ppm)

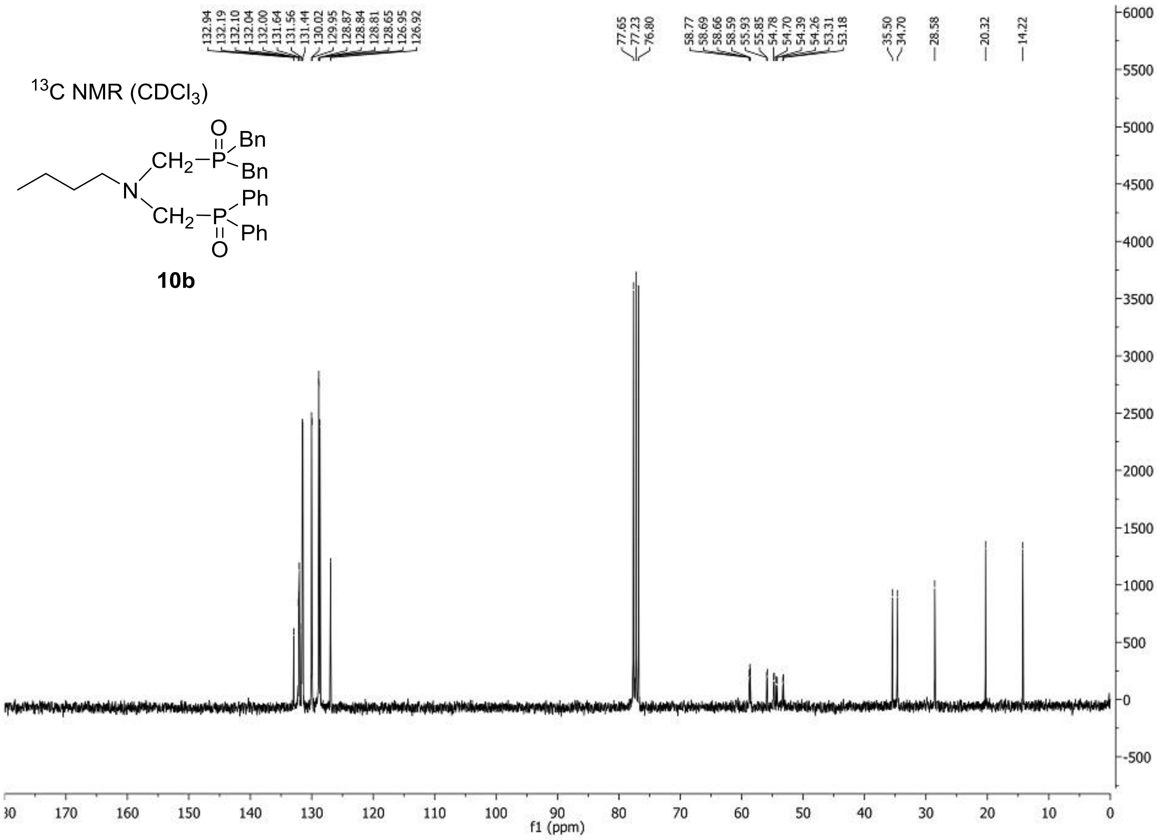
^{13}C NMR ($CDCl_3$)
10b
f1 (ppm)

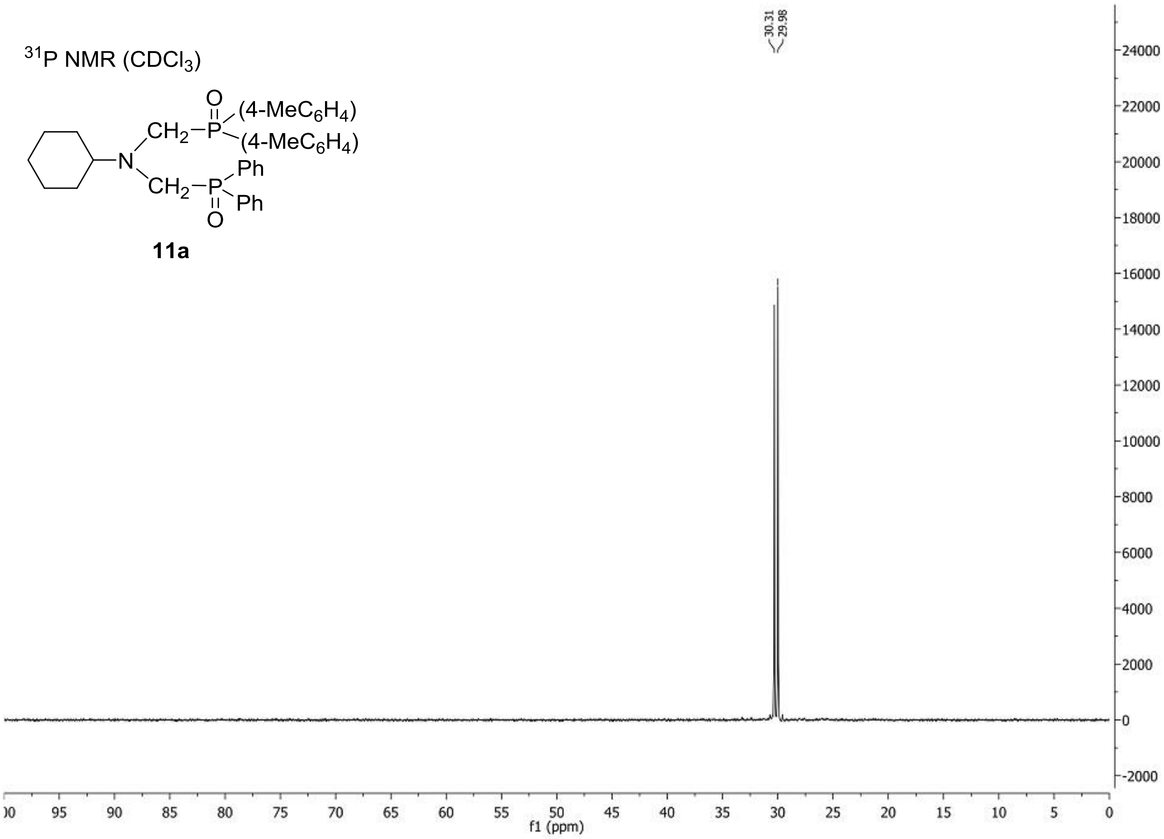
31P NMR (CDCl3)
11a
30.31
29.98
f1 (ppm)

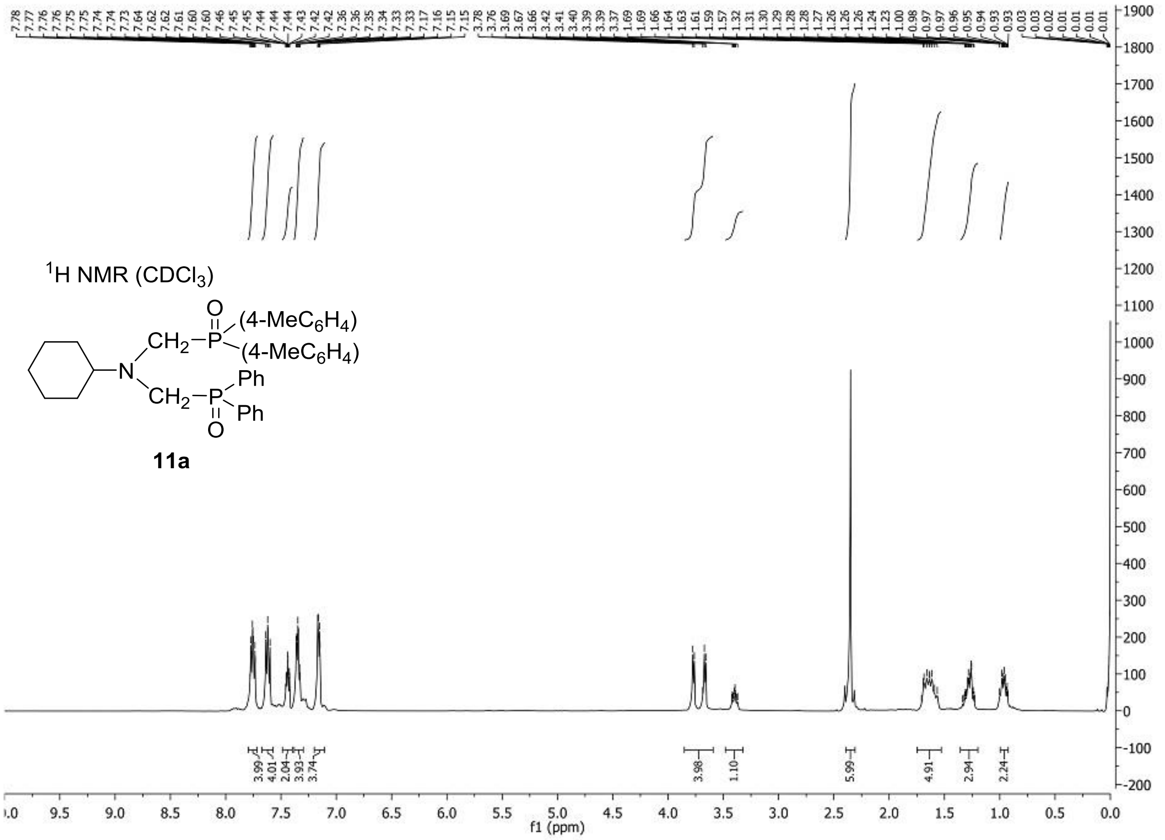
1H NMR (CDCl3)
11a
f1 (ppm)

^{13}C NMR ($CDCl_3$)

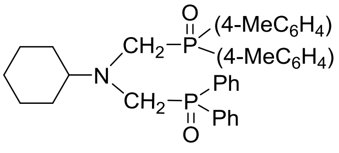

11a

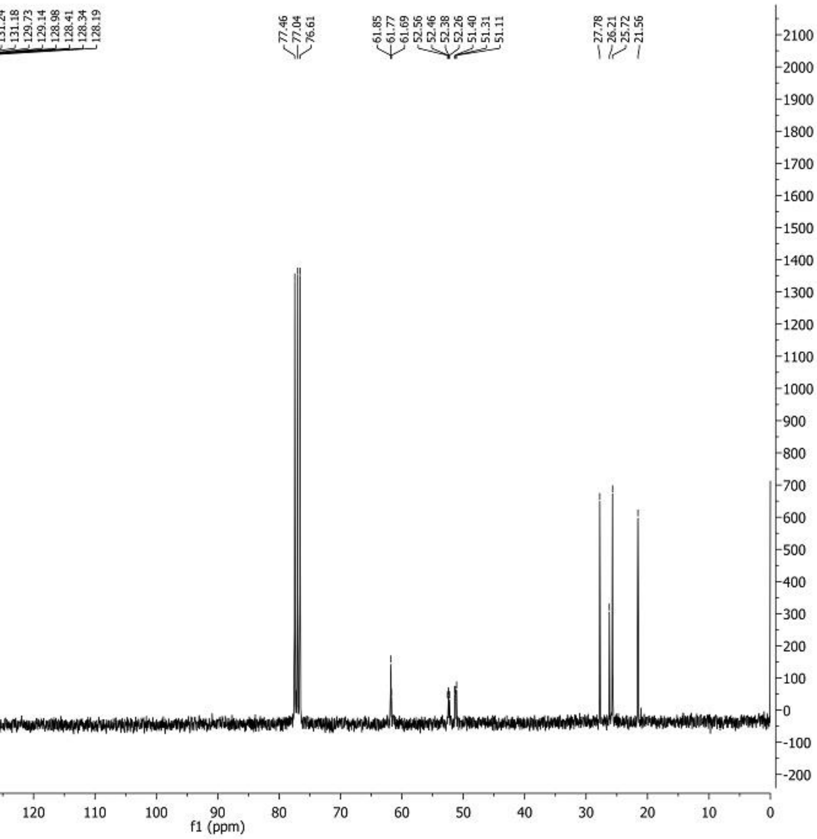

^{31}P NMR ($CDCl_3$)

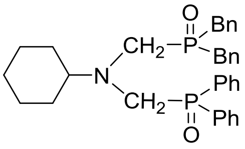

11b

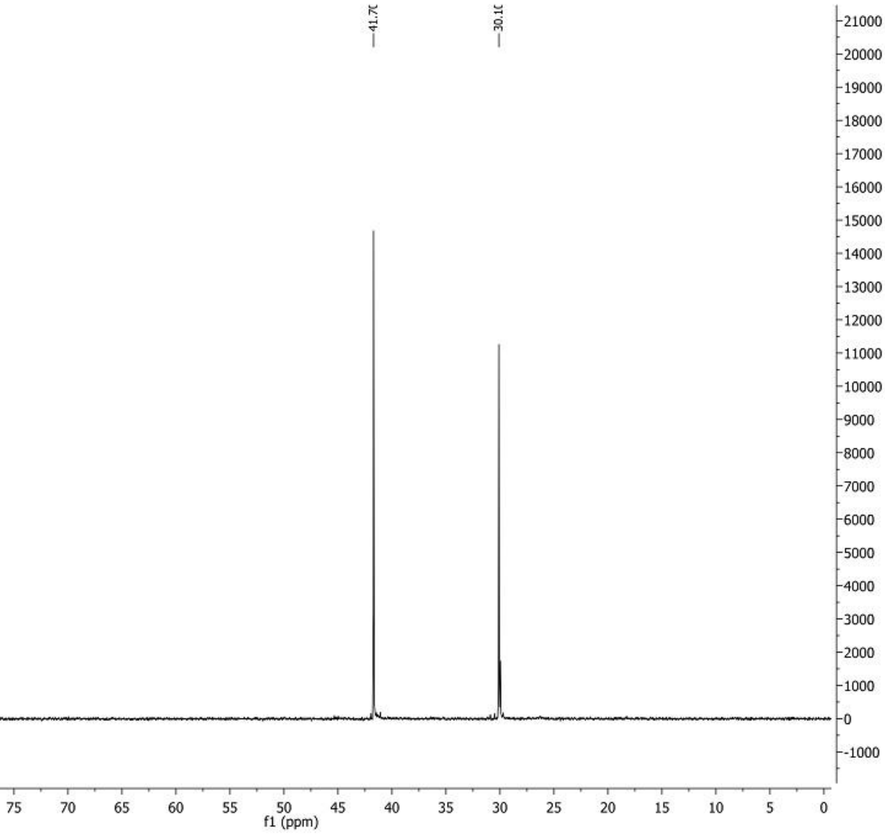

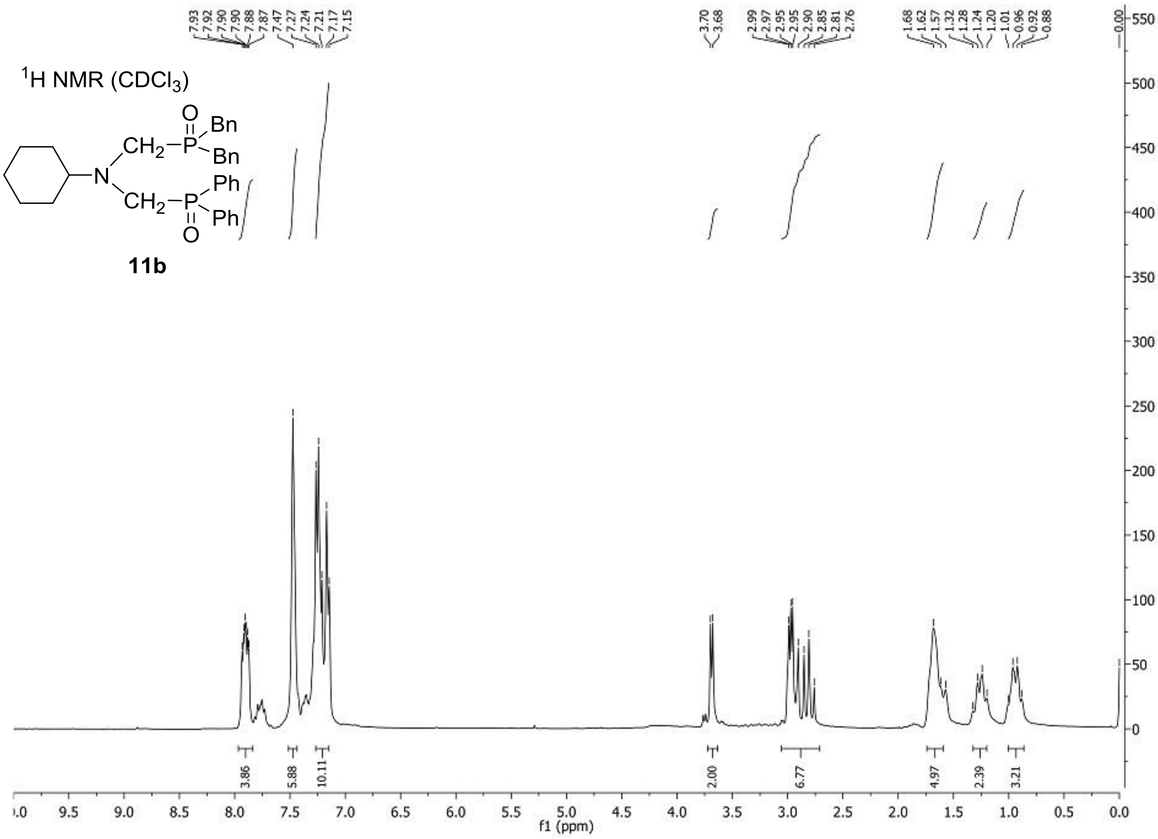

^{1}H NMR ($CDCl_3$)
11b
f1 (ppm)

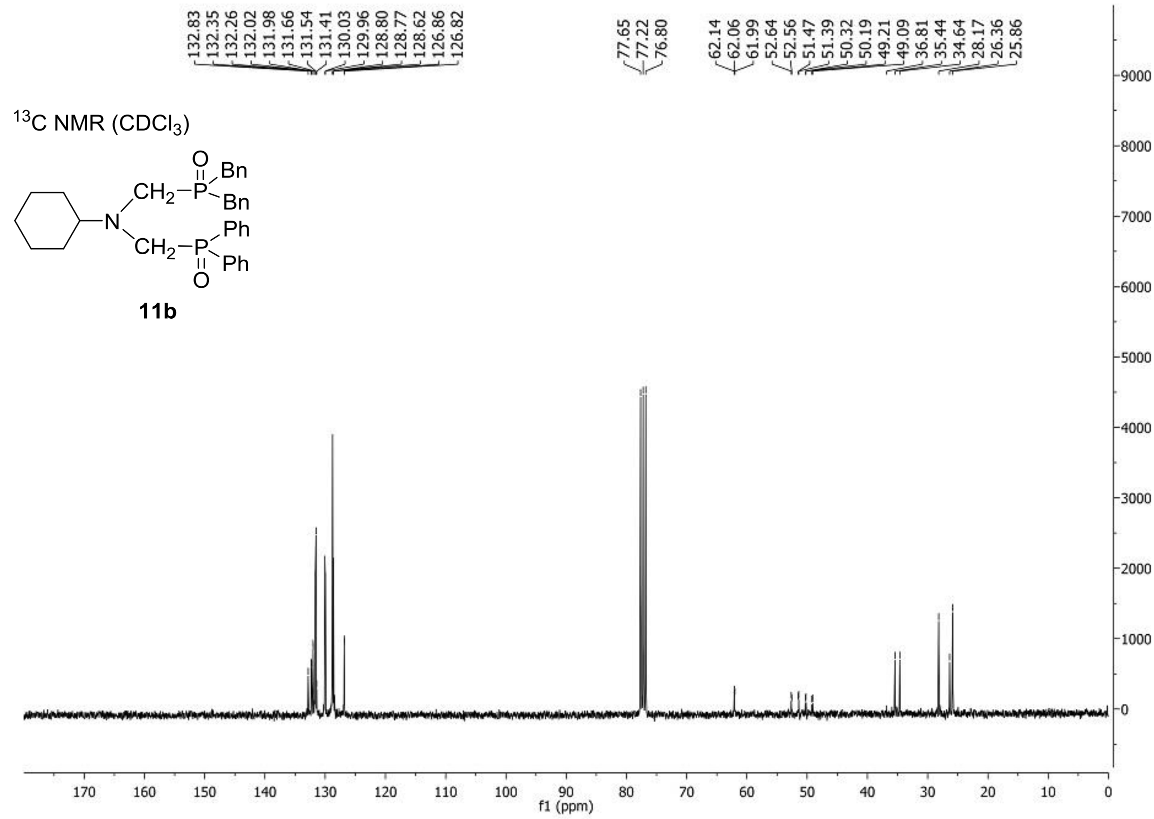

^{13}C NMR ($CDCl_3$)
11b
f1 (ppm)

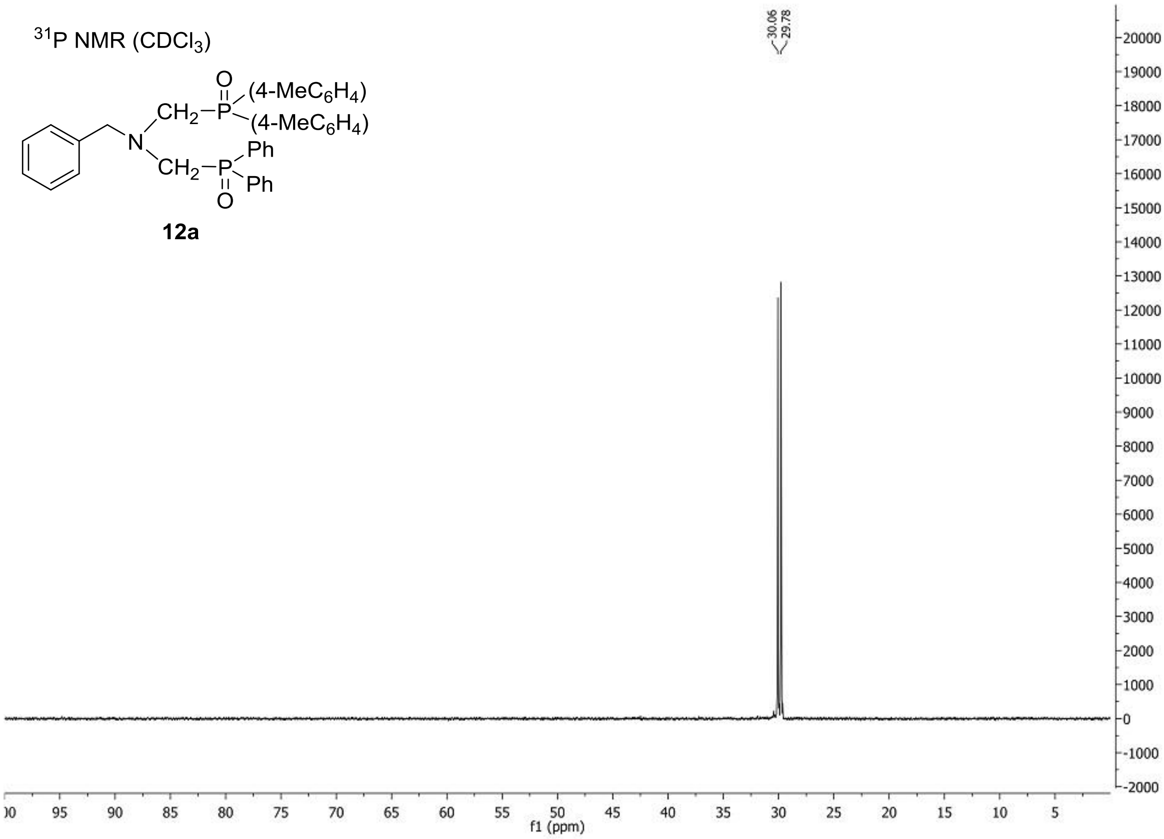

^{31}P NMR ($CDCl_3$)
(4-MeC6H4)
(4-MeC6H4)
CH2
N
CH2
Ph
Ph
12a
30.06
29.78
f1 (ppm)

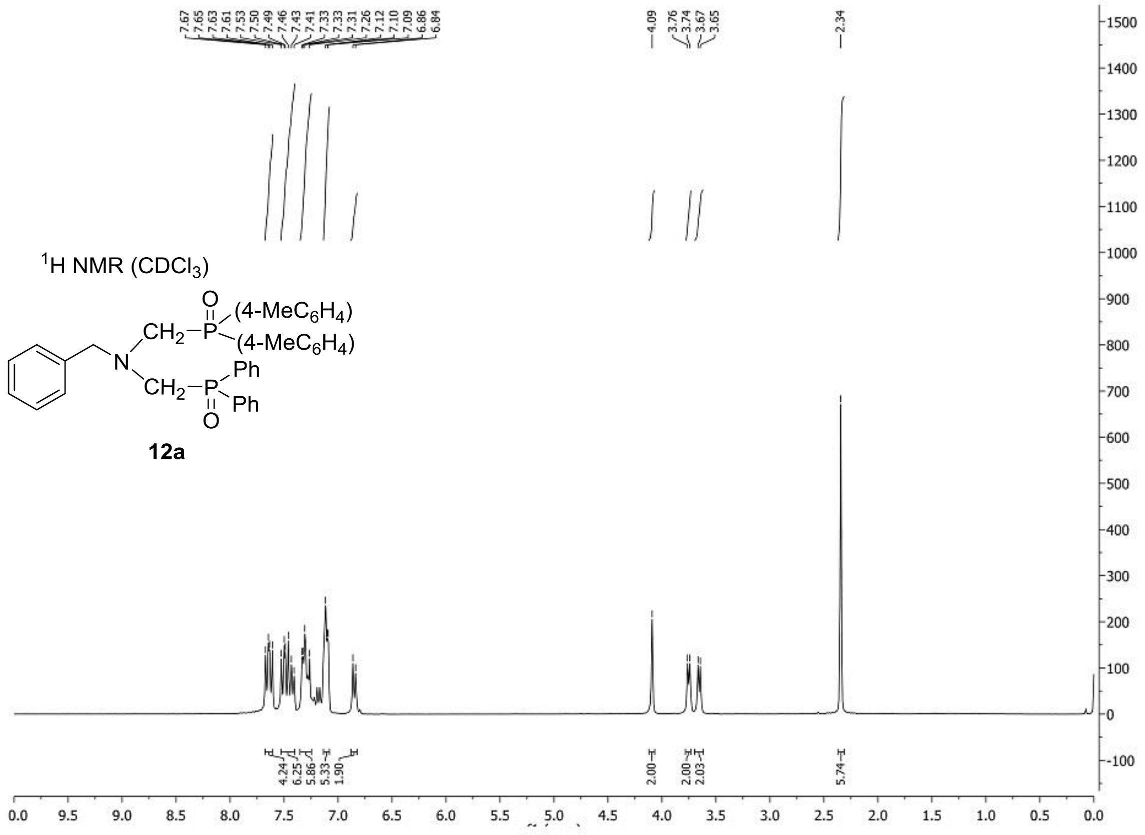

^{1}H NMR ($CDCl_3$)
(4-MeC6H4)
(4-MeC6H4)
Ph
Ph
12a
4.09
3.76
3.74
3.67
3.65
2.34
4.24
6.25
5.86
5.33
1.90
2.00
2.00
2.03
5.74

^{13}C NMR ($CDCl_3$)

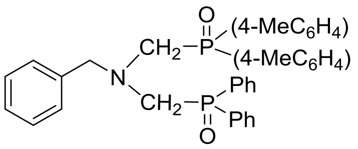

12a

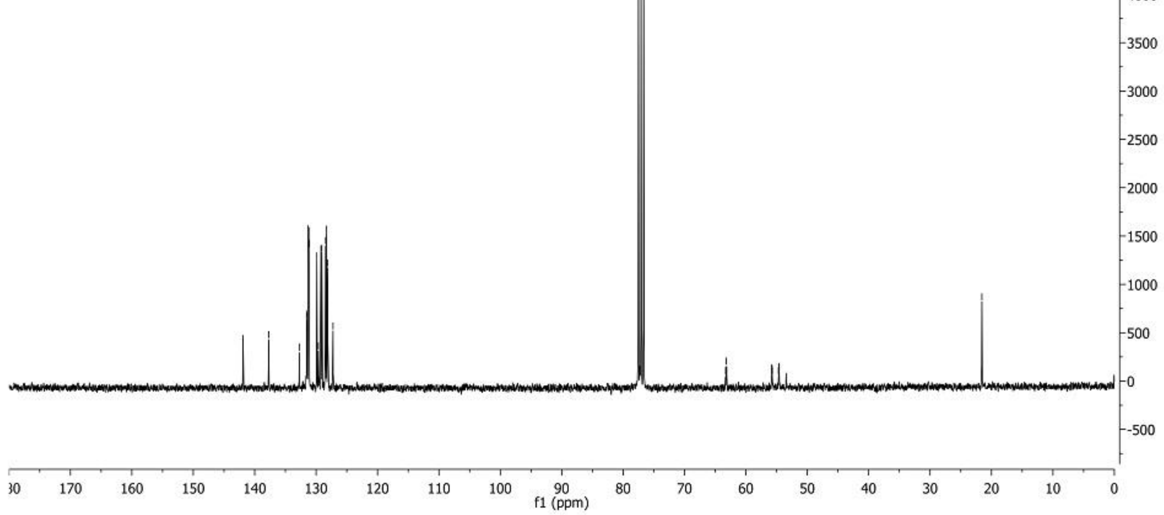

^{31}P NMR ($CDCl_3$)

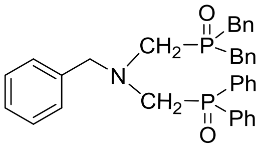

12b

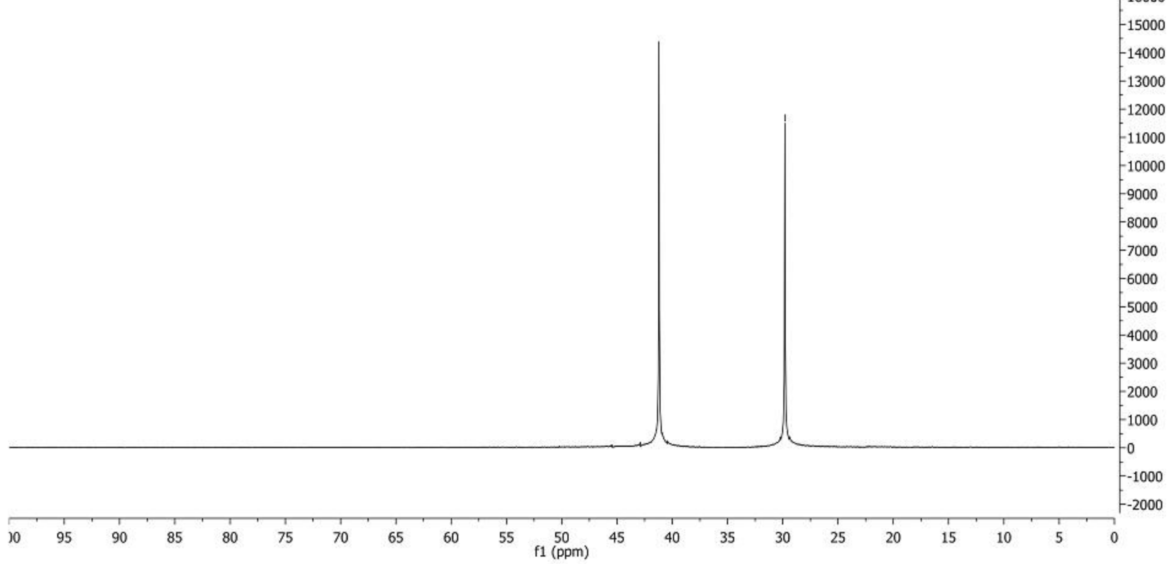

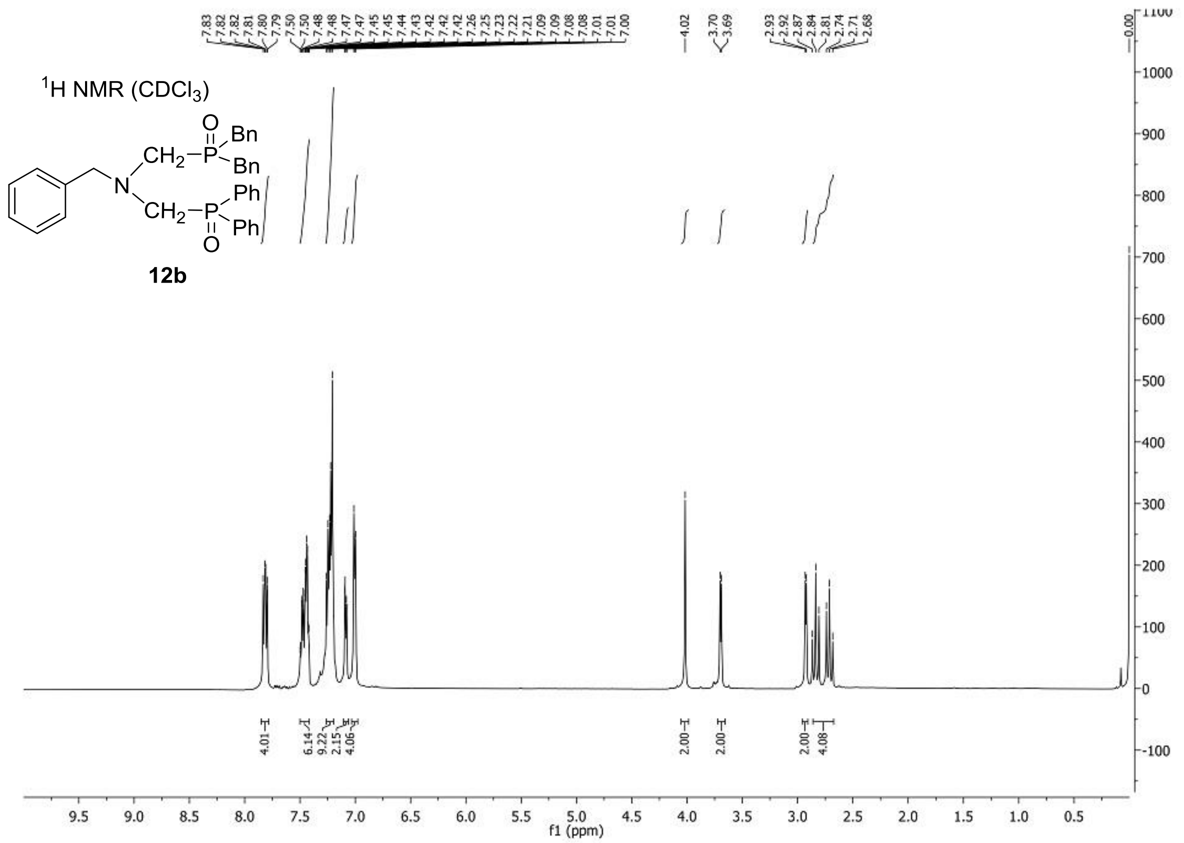
^{1}H NMR (CDCl$_3$)
12b
f1 (ppm)

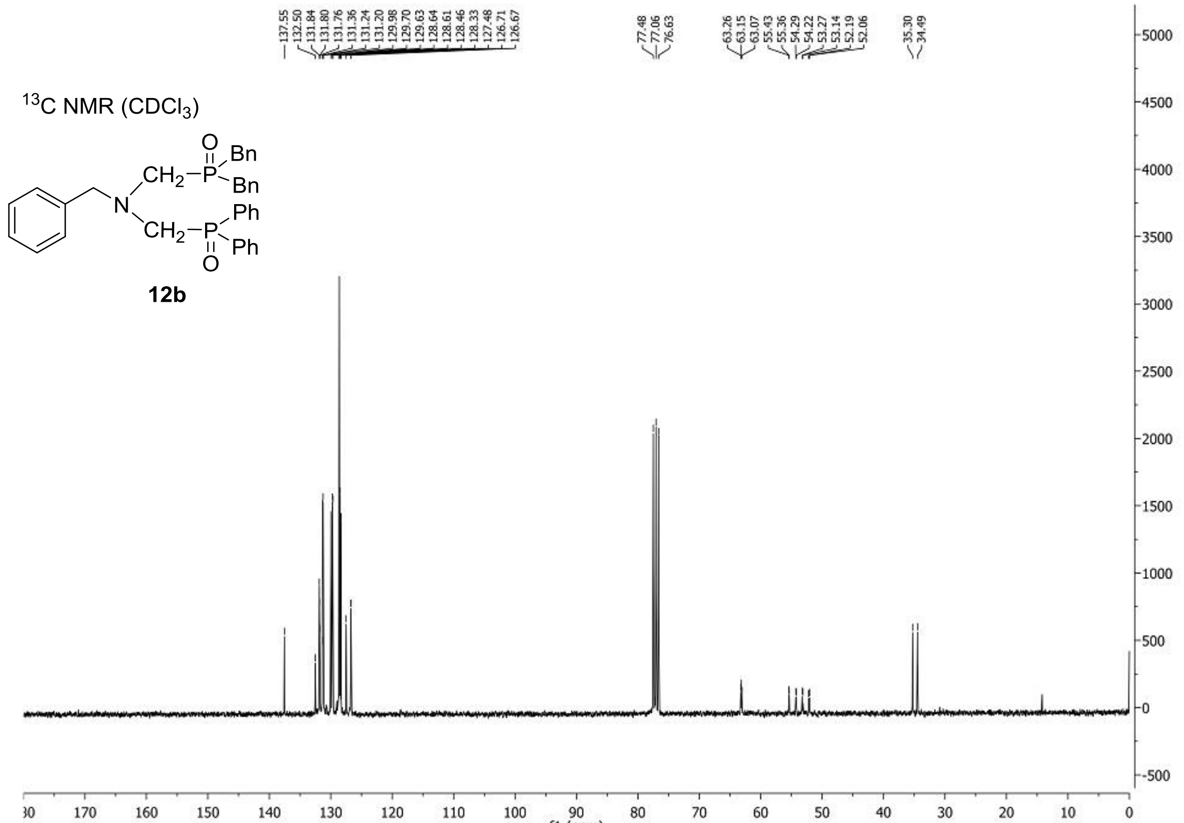
^{13}C NMR (CDCl$_3$)
12b
f1 (ppm)

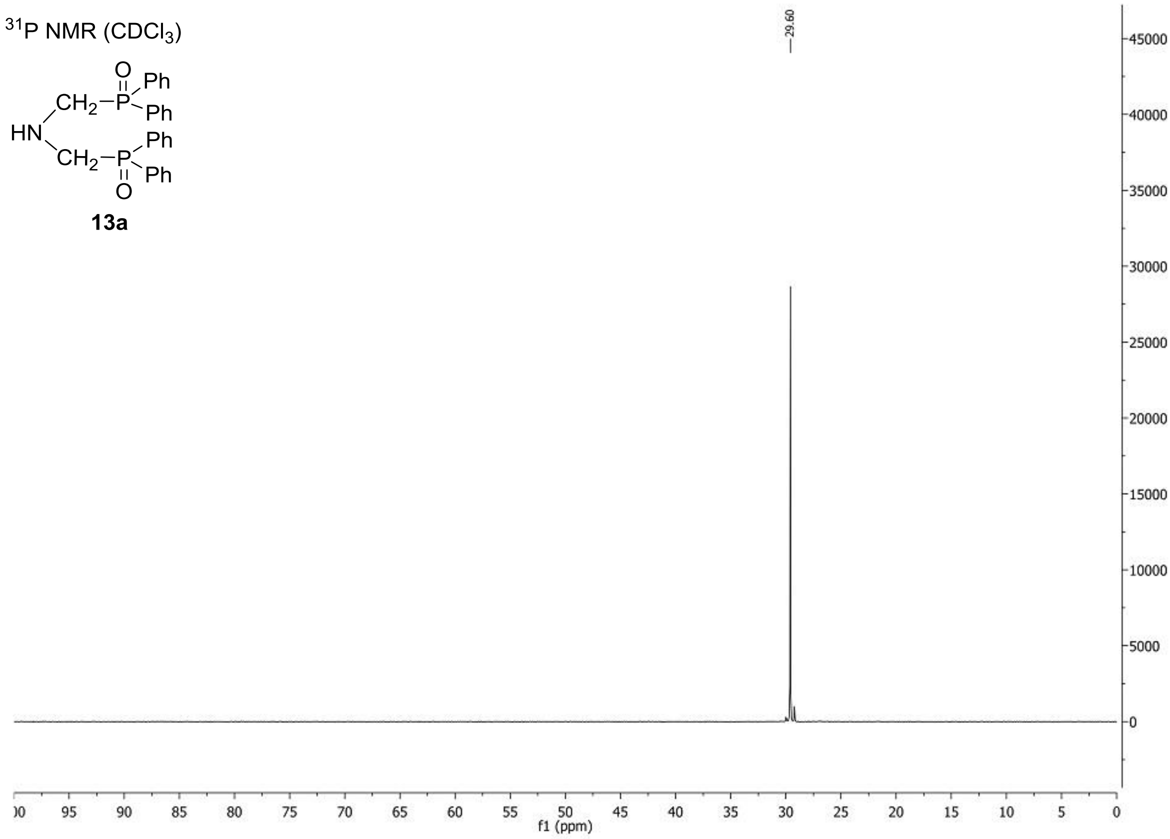

31P NMR (CDCl3)
13a
29.60
f1 (ppm)

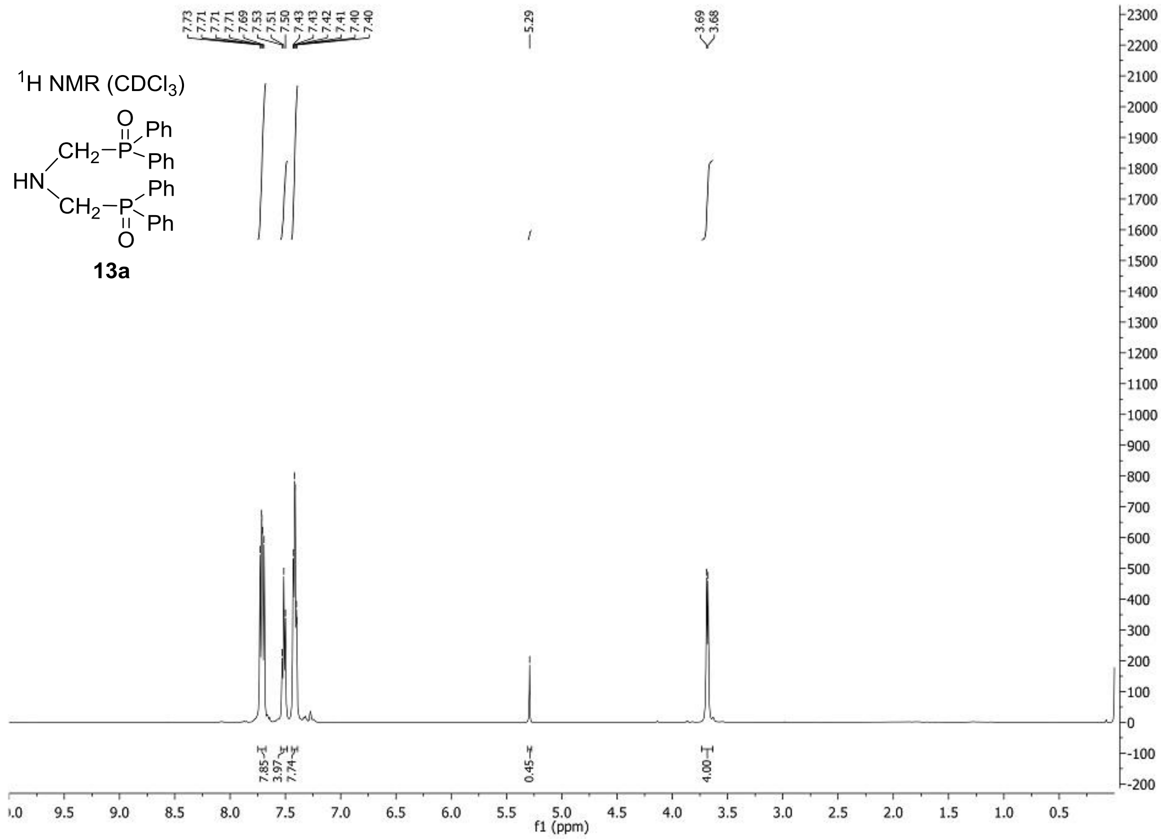

1H NMR (CDCl3)
13a
5.29
3.69
3.68
f1 (ppm)

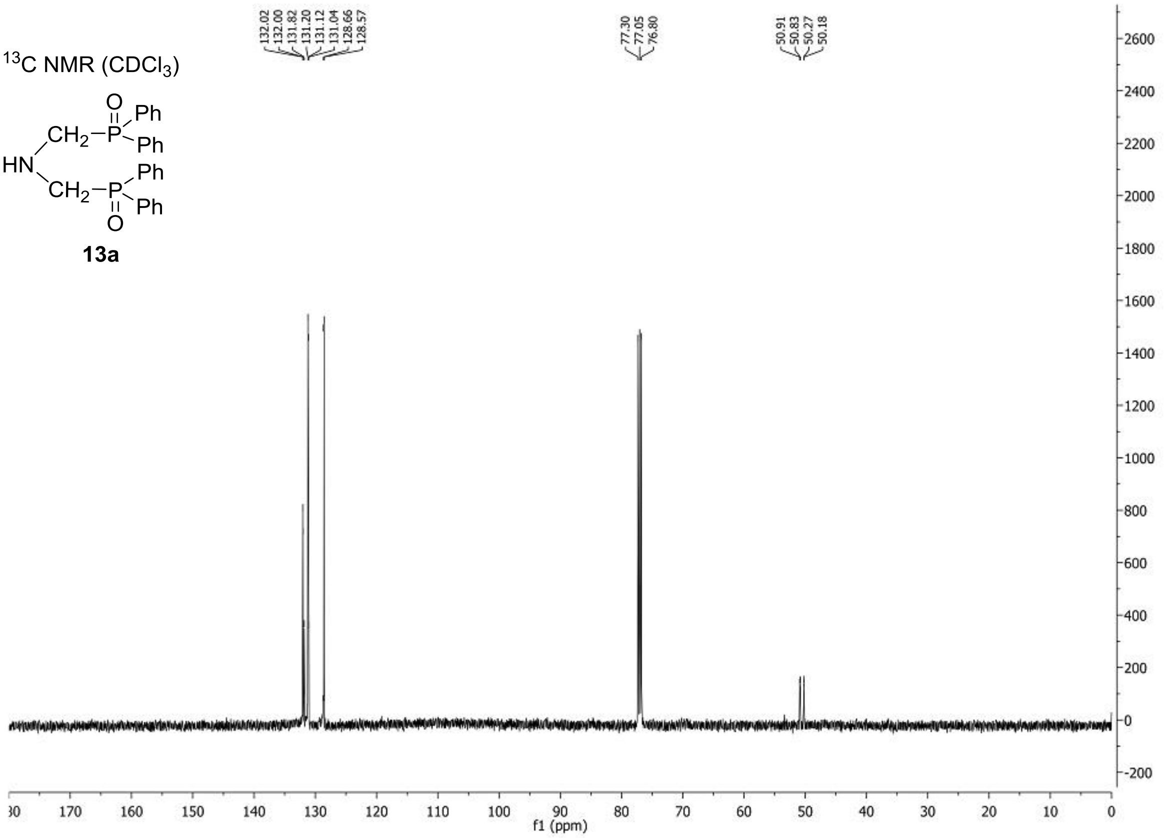
13C NMR (CDCl3)
132.02
132.00
131.82
131.20
131.12
131.04
128.66
128.57
77.30
77.05
76.80
50.91
50.83
50.27
50.18
f1 (ppm)

13a

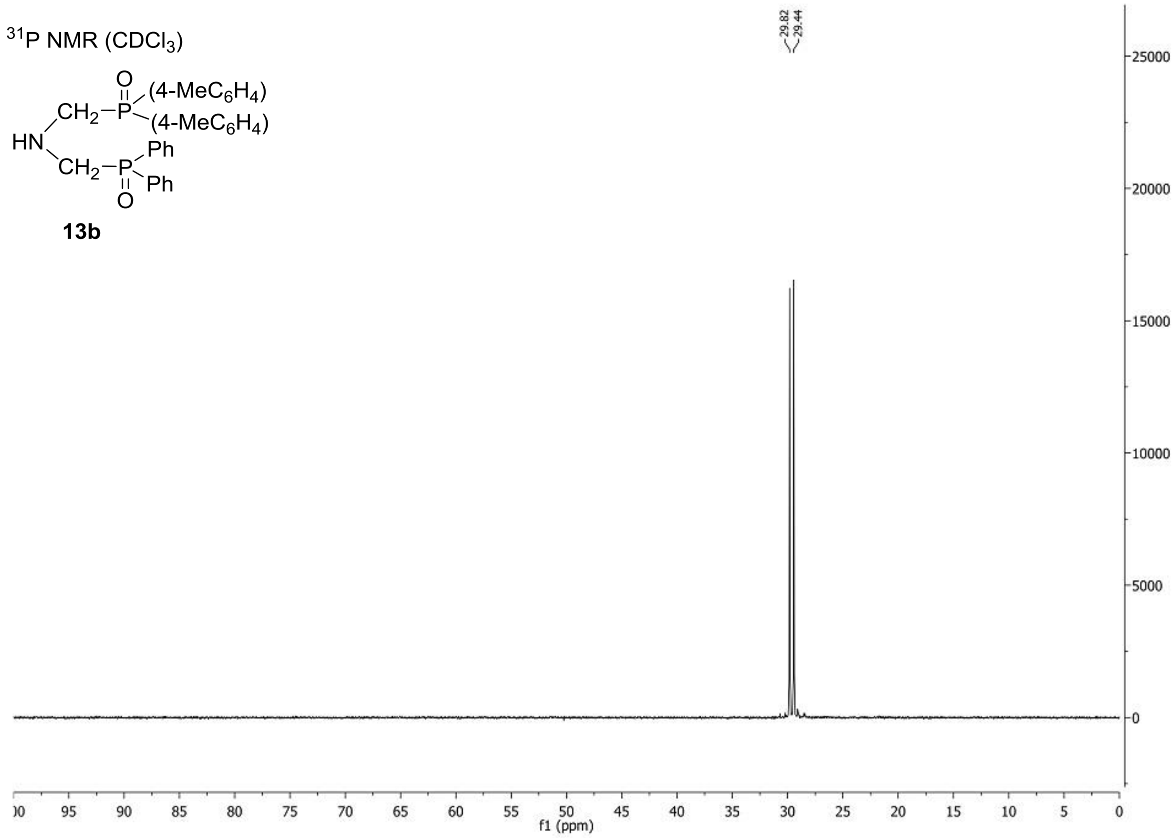
31P NMR (CDCl3)
29.82
29.44
f1 (ppm)

13b

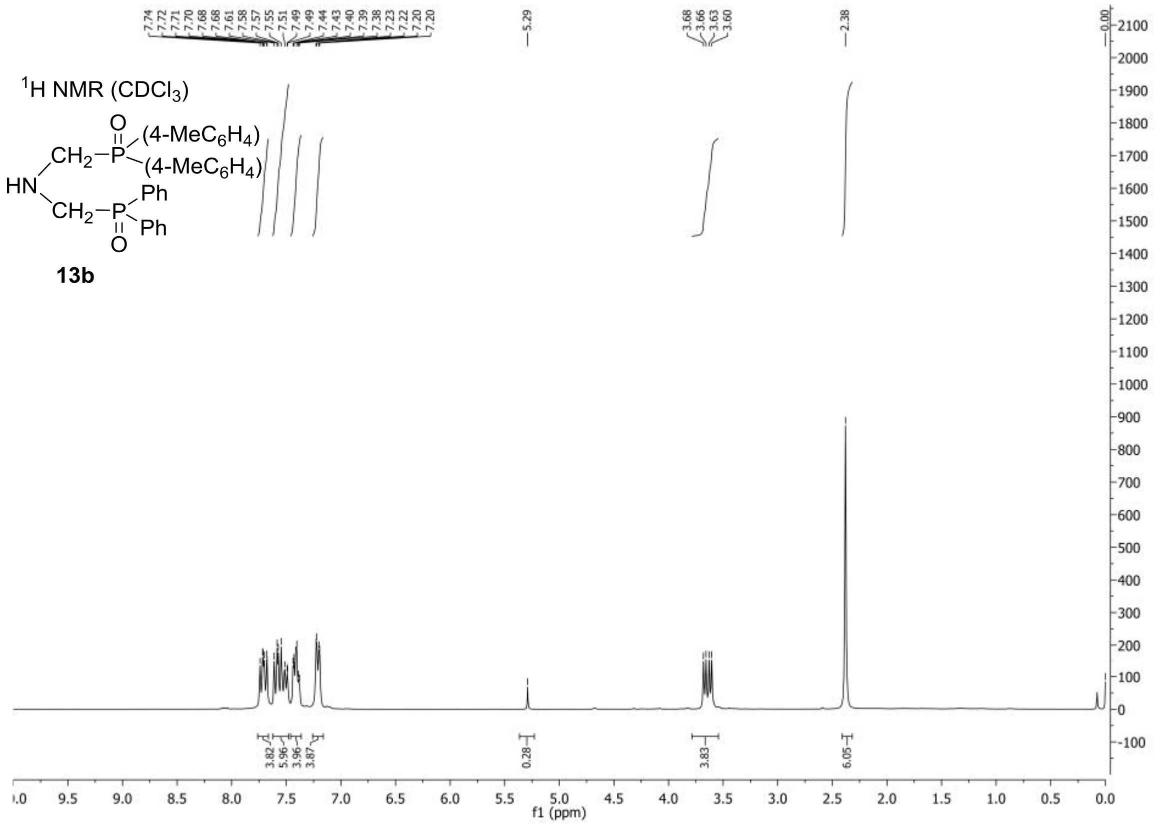

^{1}H NMR (CDCl$_3$)
13b
f1 (ppm)

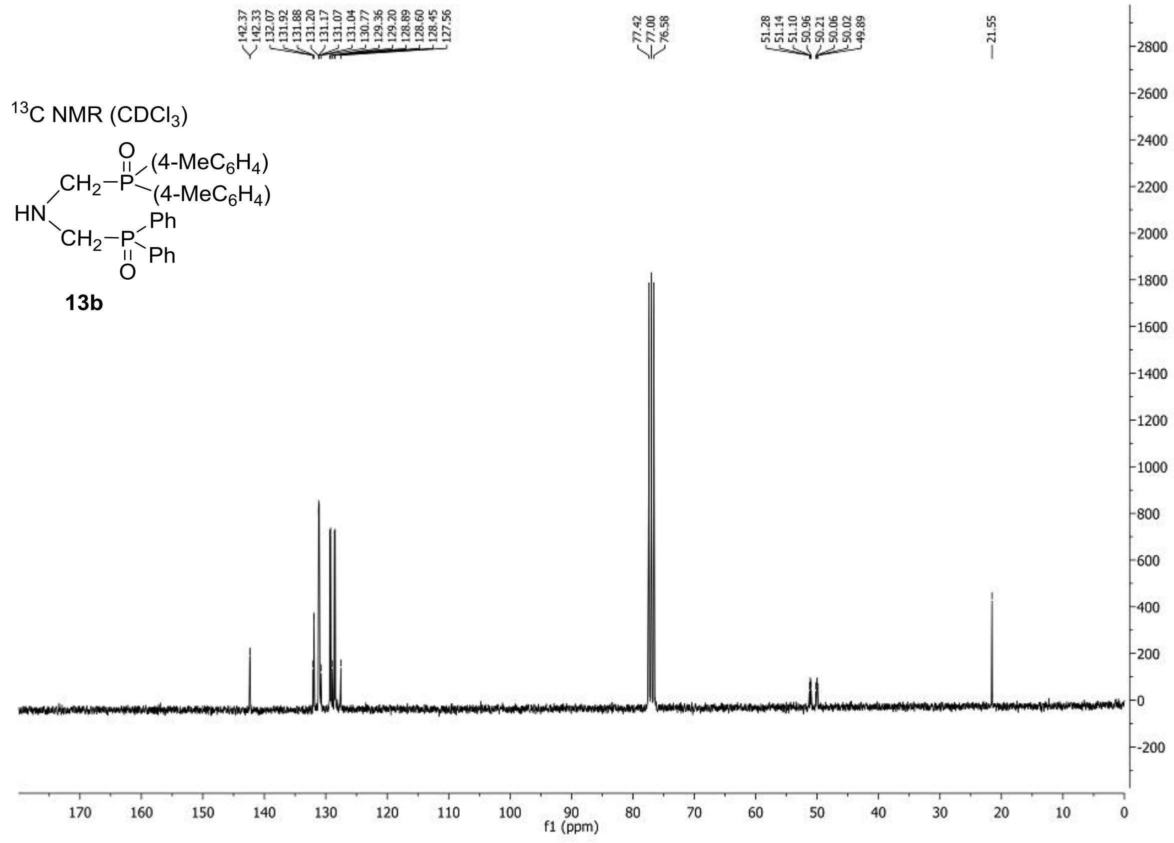

^{13}C NMR (CDCl$_3$)
13b
f1 (ppm)

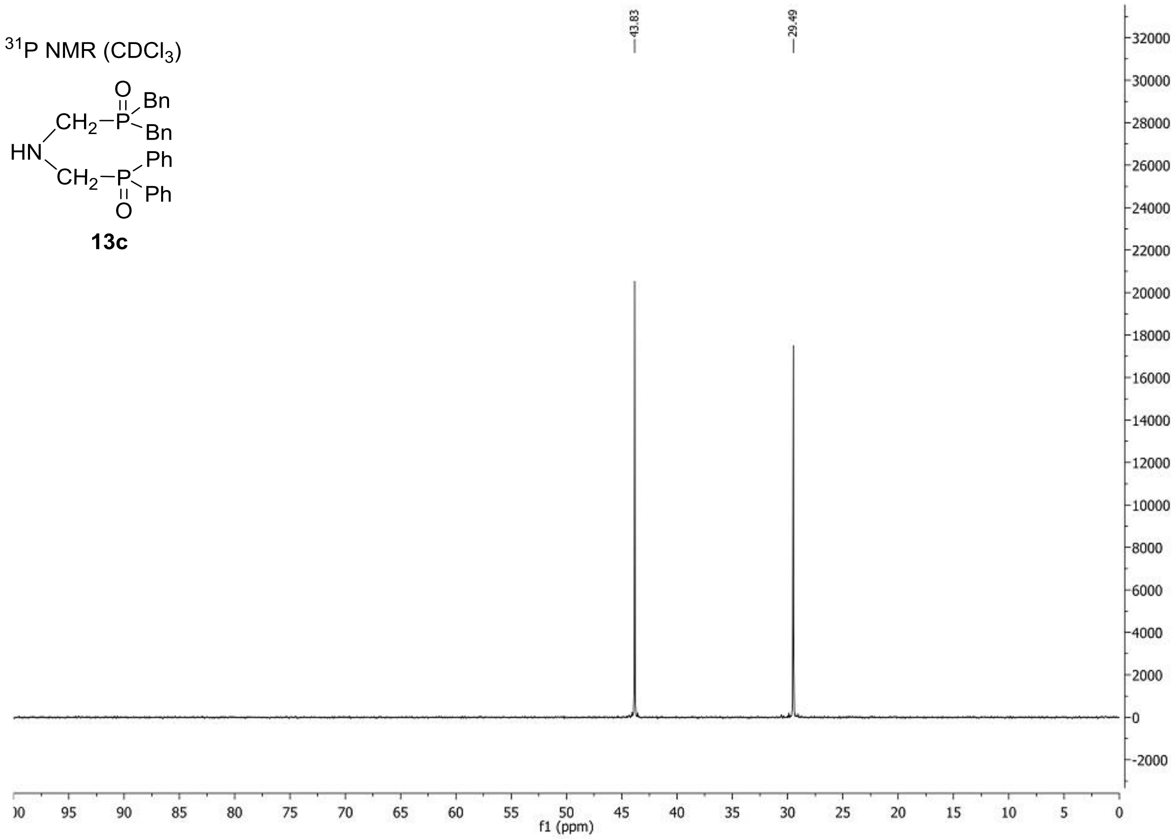

31P NMR (CDCl3)
13c
43.83
29.49
f1 (ppm)

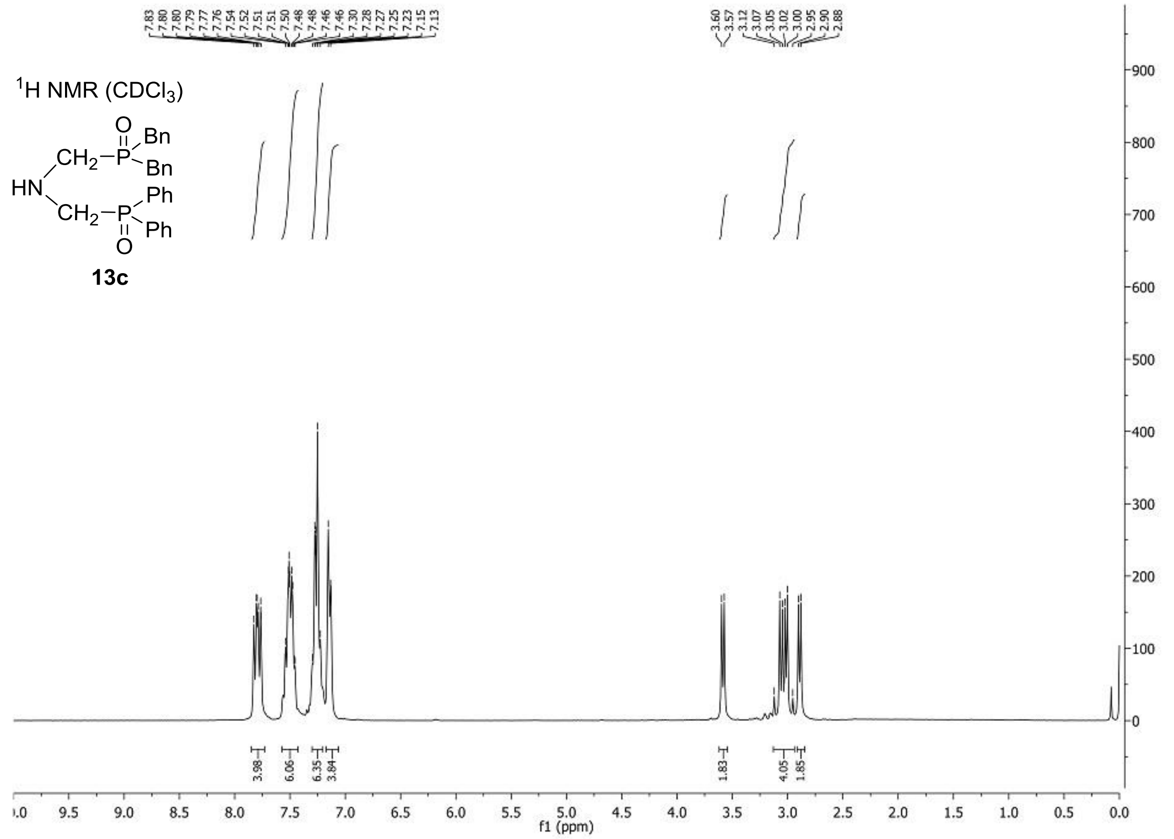

1H NMR (CDCl3)
13c
7.83 7.80 7.80 7.79 7.77 7.76 7.54 7.52 7.51 7.51 7.50 7.48 7.48 7.46 7.46 7.30 7.28 7.27 7.25 7.23 7.15 7.13
3.60 3.57 3.12 3.07 3.05 3.02 3.00 2.95 2.90 2.88
3.98 6.06 6.35 3.84 1.83 4.05 1.85
f1 (ppm)

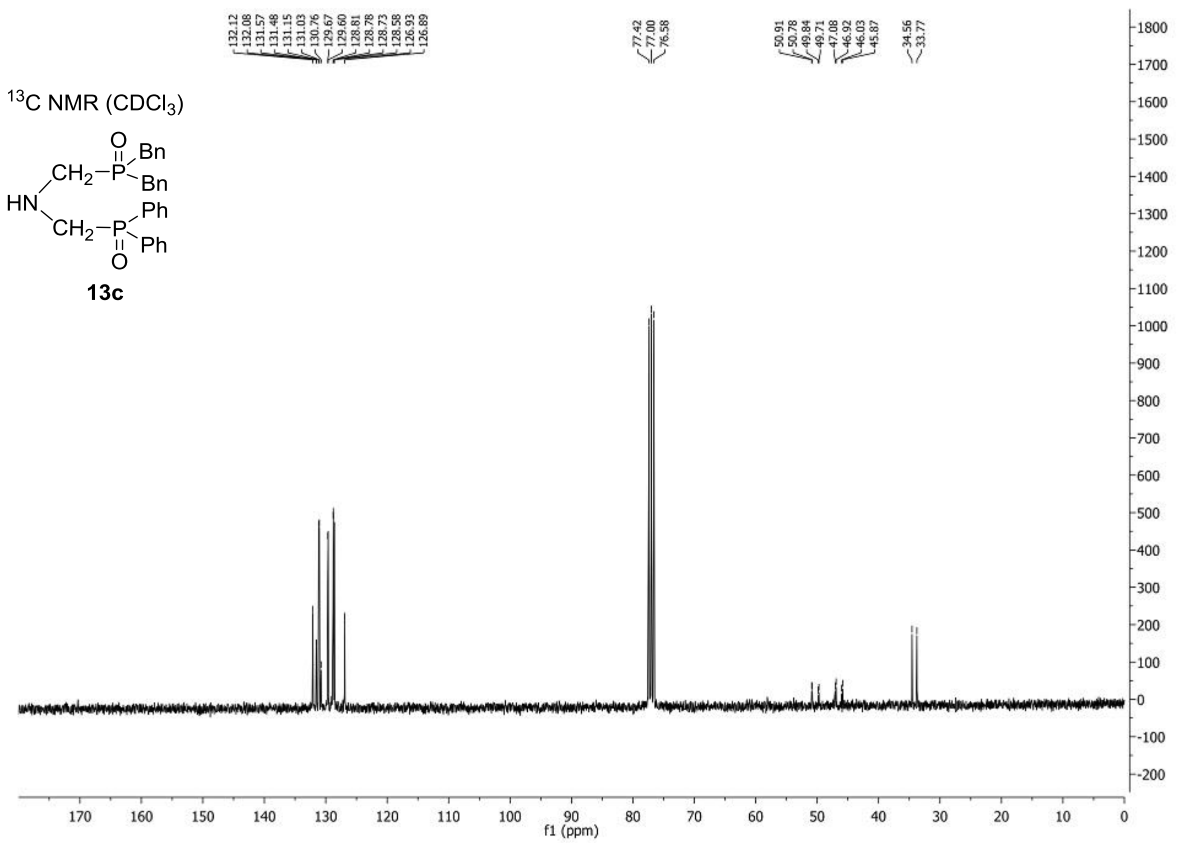
13C NMR (CDCl3)
13c
132.12 132.08 131.57 131.48 131.15 131.03 130.76 129.67 129.60 128.81 128.78 128.73 128.58 126.93 126.89
77.42 77.00 76.58
50.91 50.78 49.84 49.71 47.08 46.92 46.03 45.87
34.56 33.77
f1 (ppm)

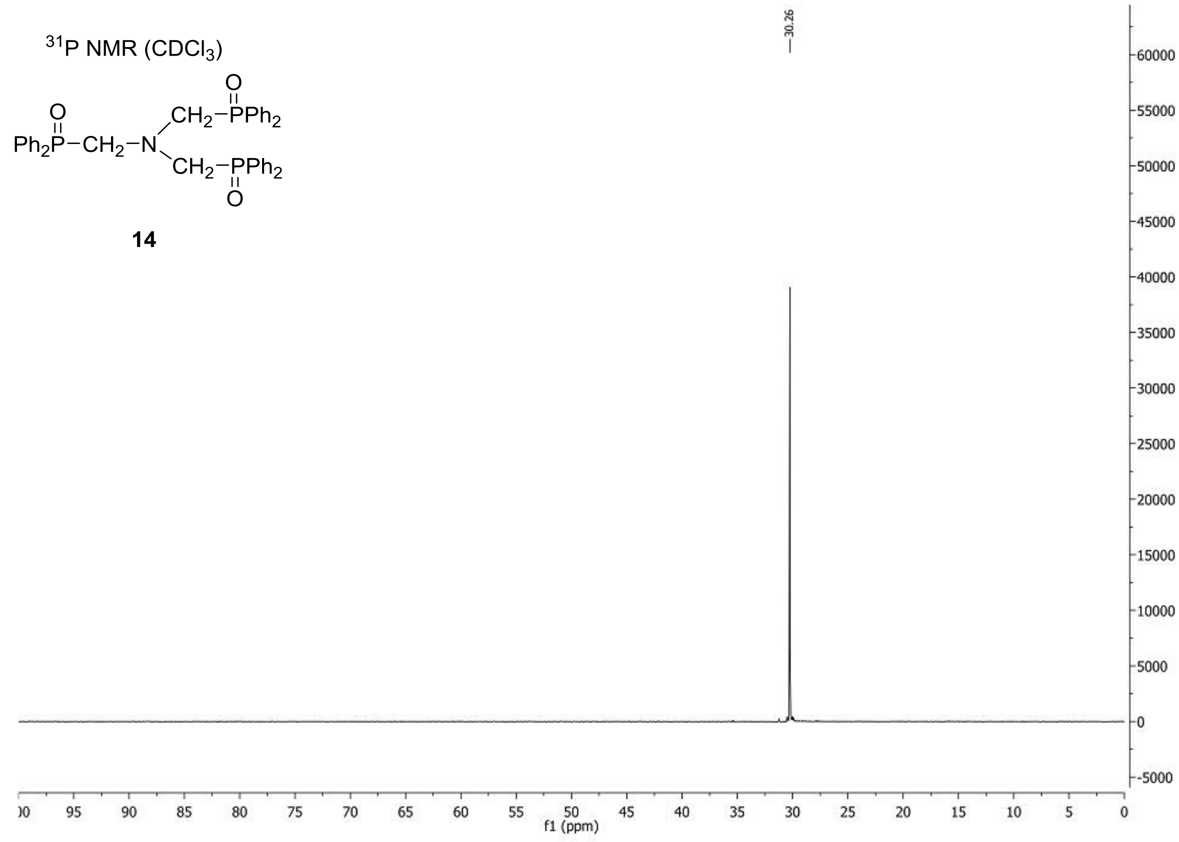
31P NMR (CDCl3)
14
30.26
f1 (ppm)

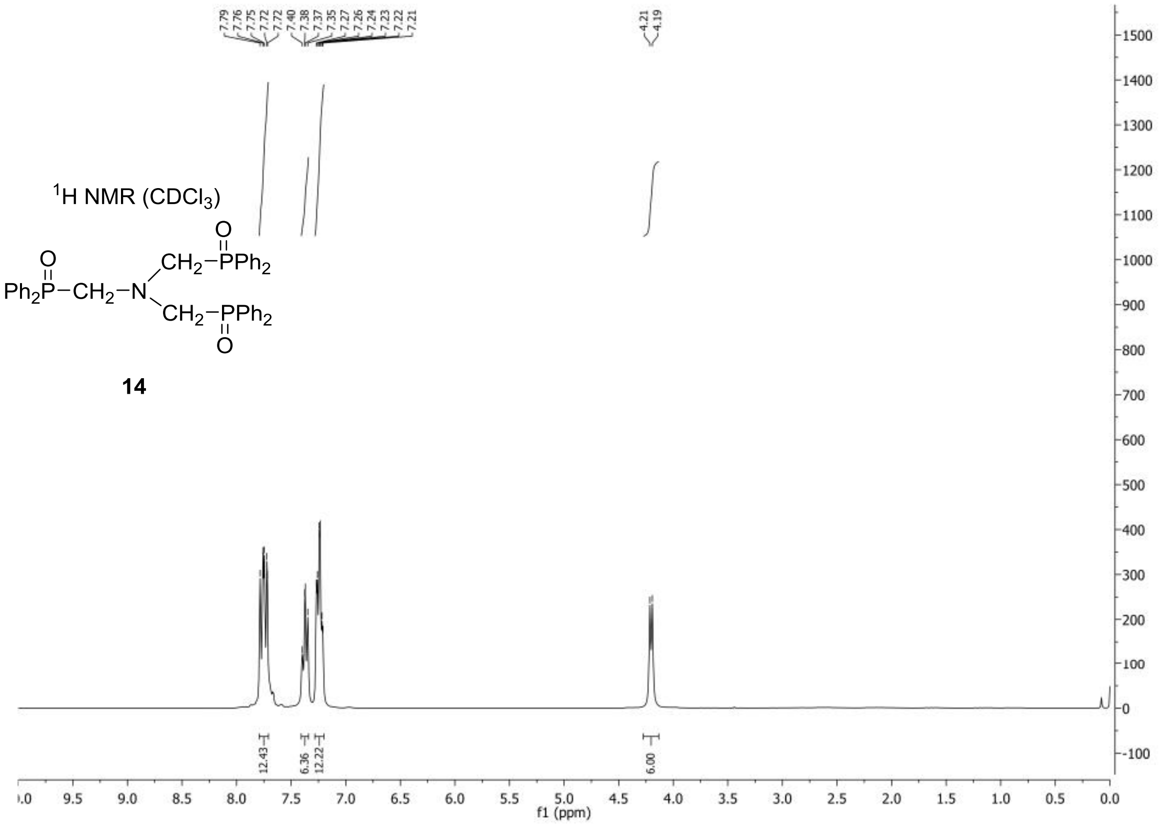

^{1}H NMR (CDCl$_3$)
14
f1 (ppm)

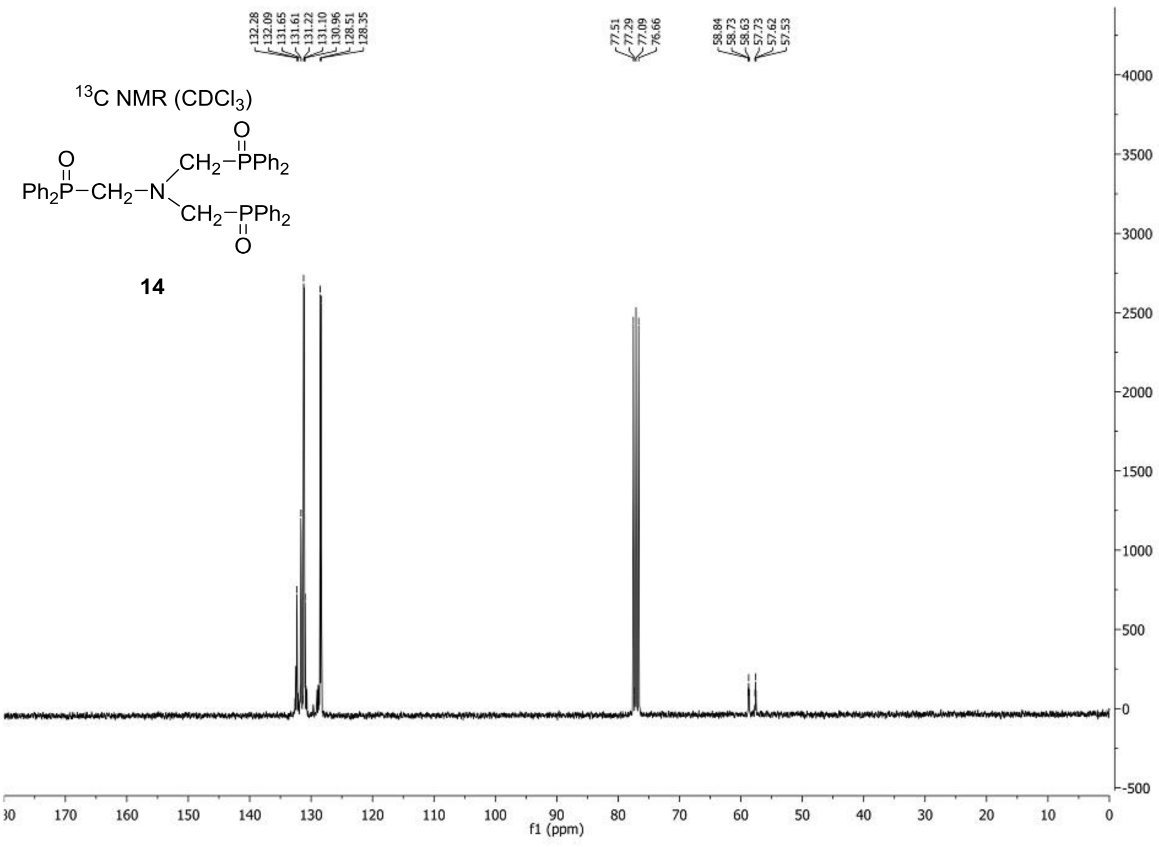

^{13}C NMR (CDCl$_3$)
14
f1 (ppm)

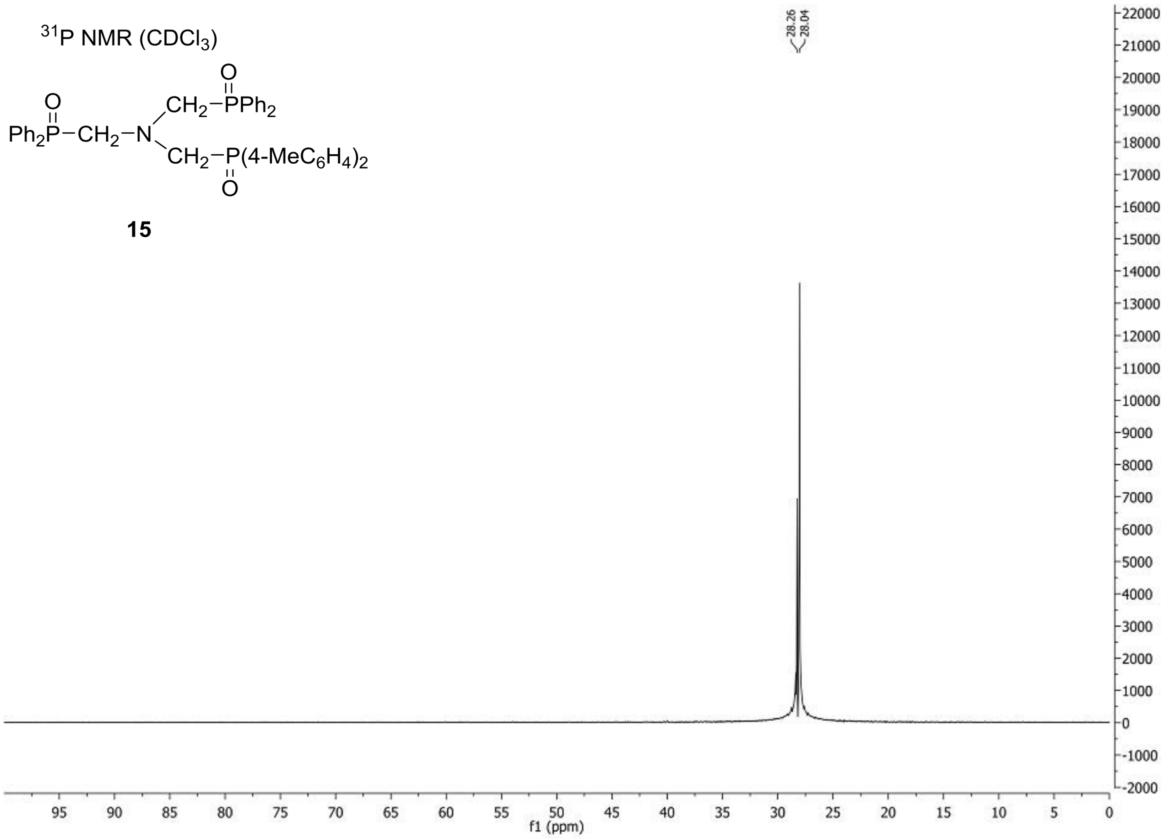

^{31}P NMR ($CDCl_3$)
15
28.26
28.04
f1 (ppm)

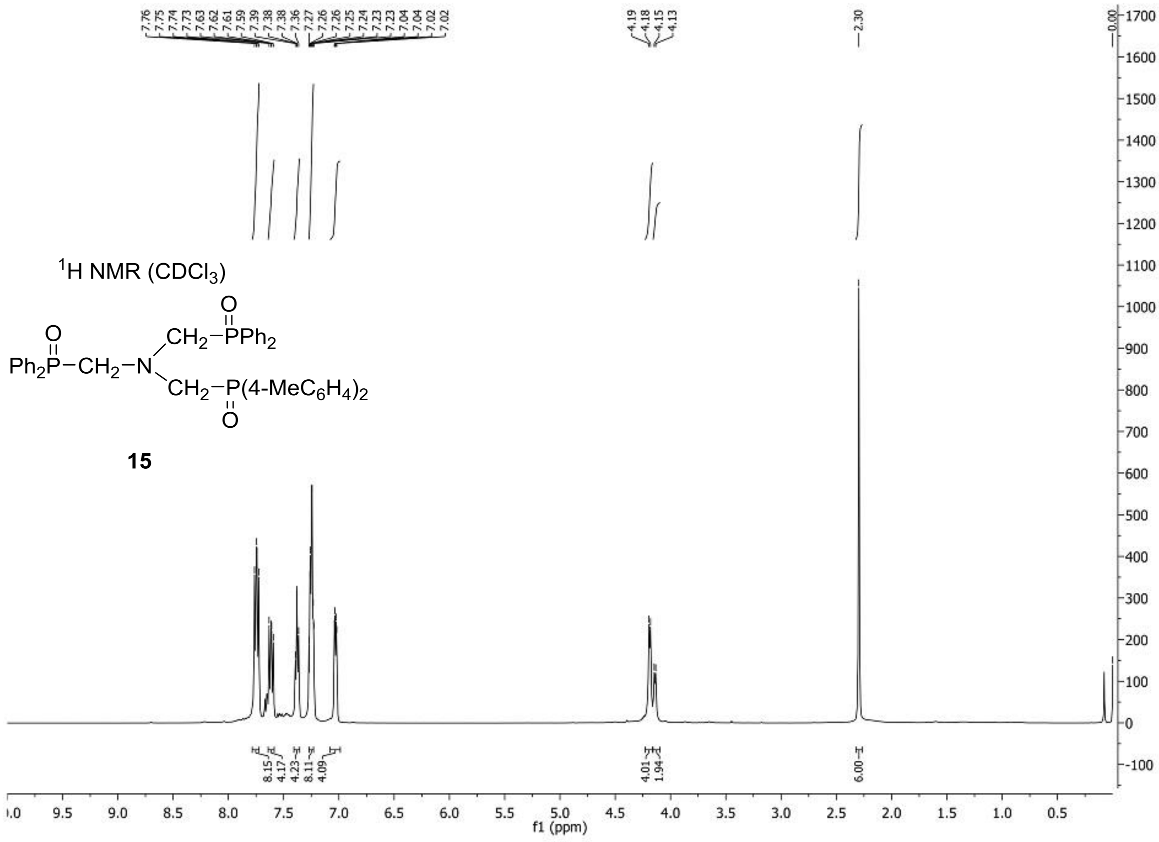

^{1}H NMR ($CDCl_3$)
15
7.76 7.75 7.74 7.73 7.63 7.62 7.61 7.59 7.39 7.38 7.38 7.36 7.27 7.26 7.26 7.25 7.24 7.23 7.23 7.04 7.04 7.02 7.02
4.19 4.18 4.15 4.13
2.30
0.00
8.15 4.17 4.23 8.11 4.09
4.01 1.94
6.00
f1 (ppm)

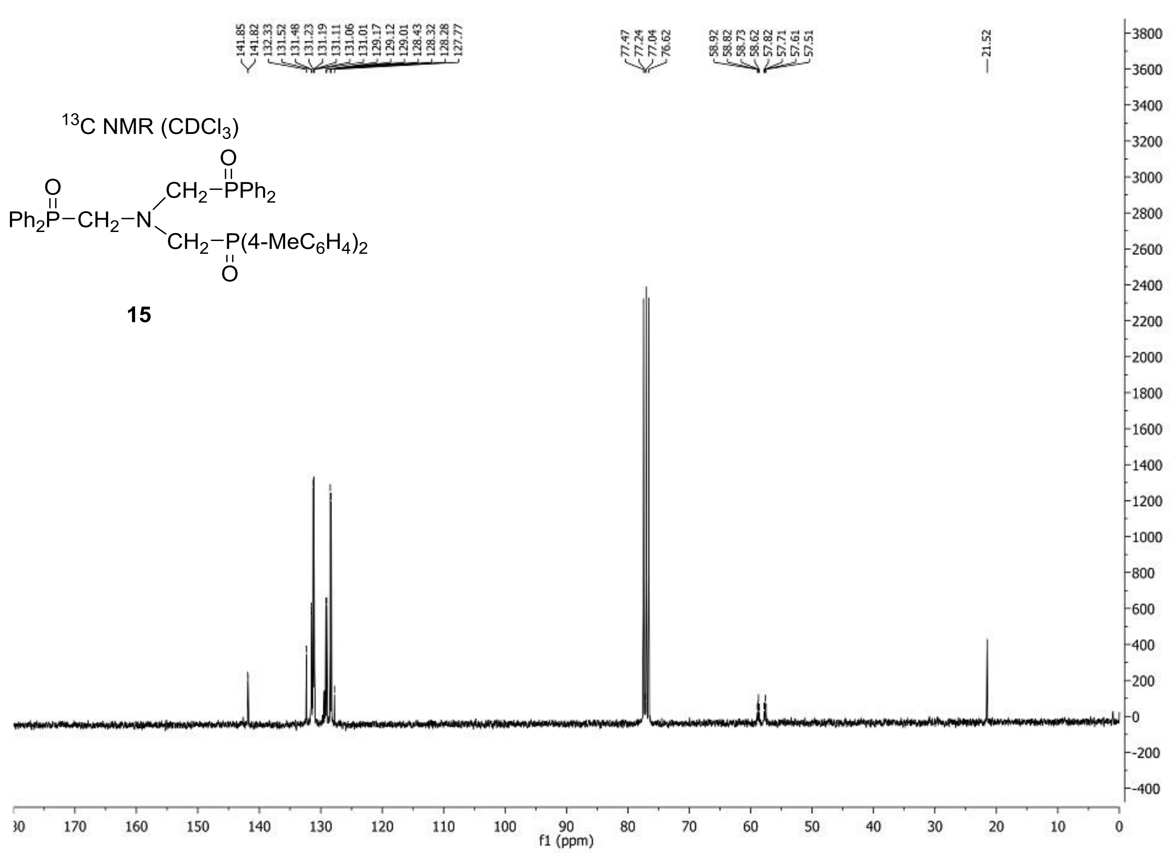

13C NMR (CDCl3)
Ph2P(O)–CH2–N(CH2–P(O)Ph2)(CH2–P(O)(4-MeC6H4)2)
15
141.85 141.82 132.33 131.52 131.48 131.23 131.19 131.11 131.06 131.01 129.17 129.12 129.01 128.43 128.32 128.28 127.77
77.47 77.24 77.04 76.62
58.92 58.82 58.73 58.62 57.82 57.71 57.61 57.51
21.52
f1 (ppm)

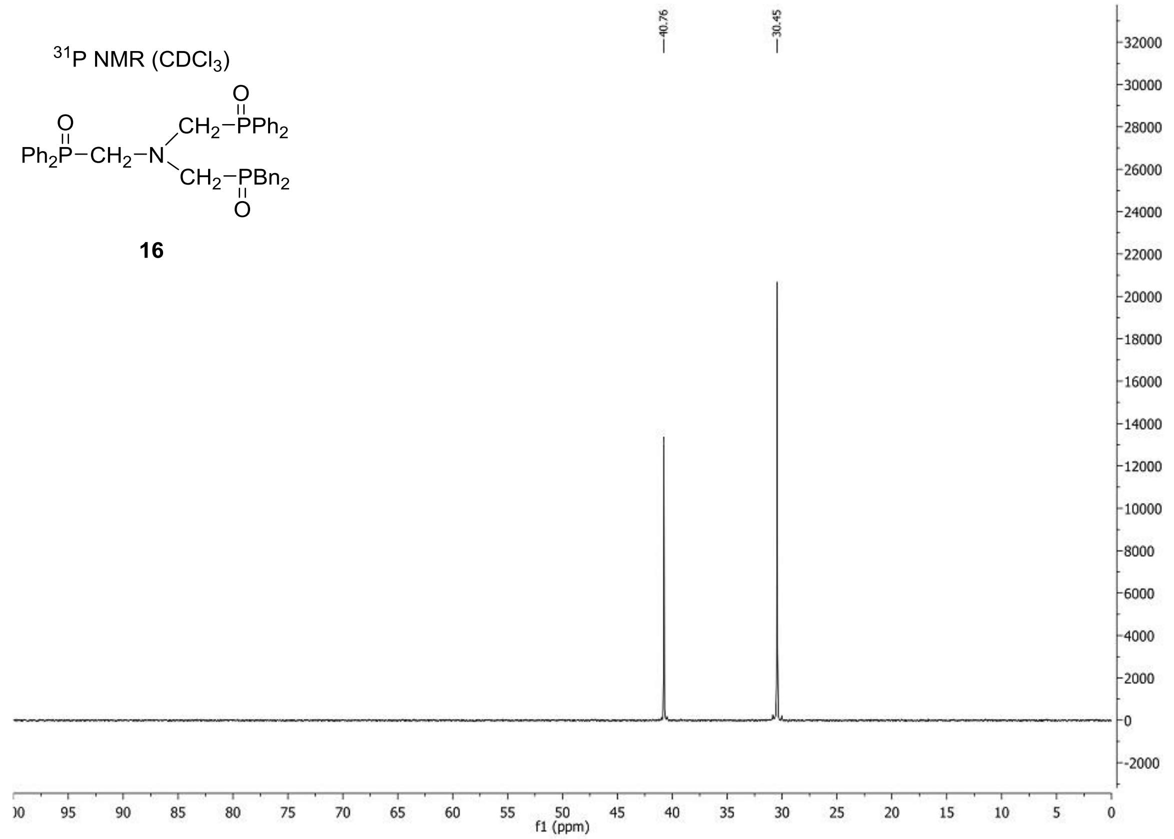

31P NMR (CDCl3)
Ph2P(O)–CH2–N(CH2–P(O)Ph2)(CH2–P(O)Bn2)
16
40.76
30.45
f1 (ppm)

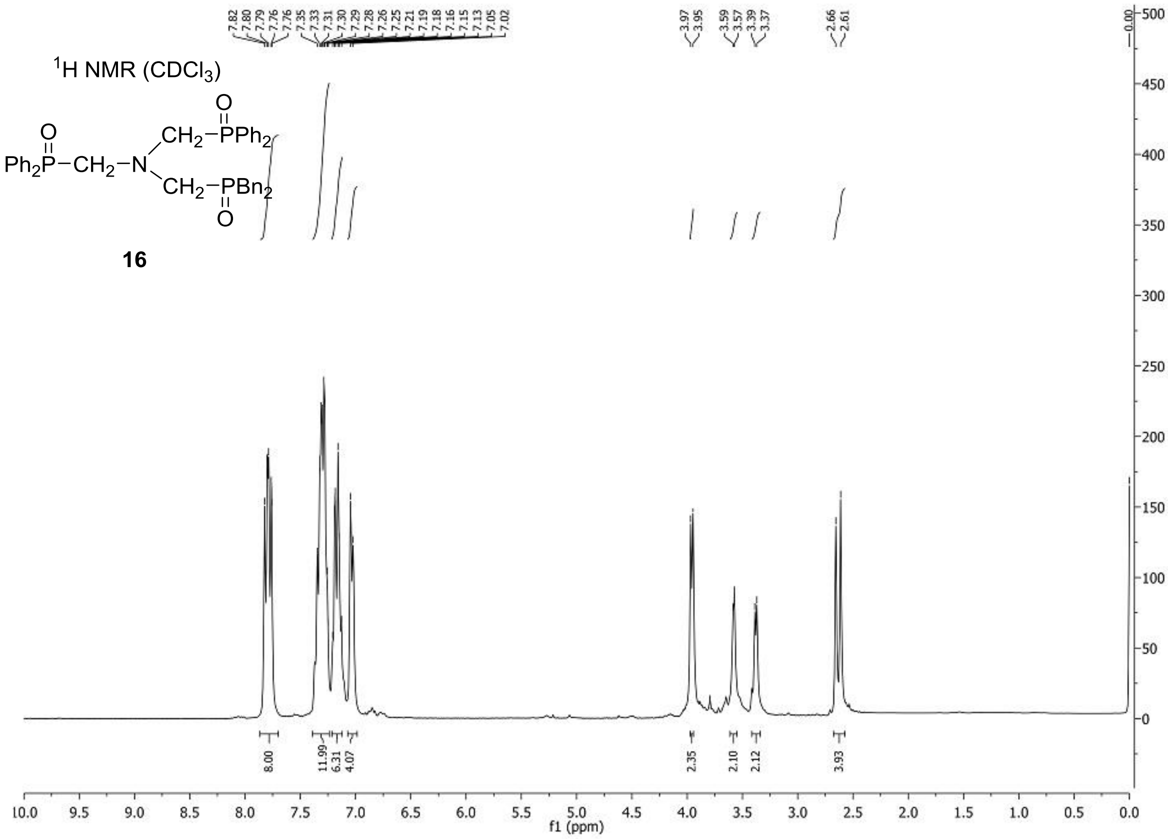

16

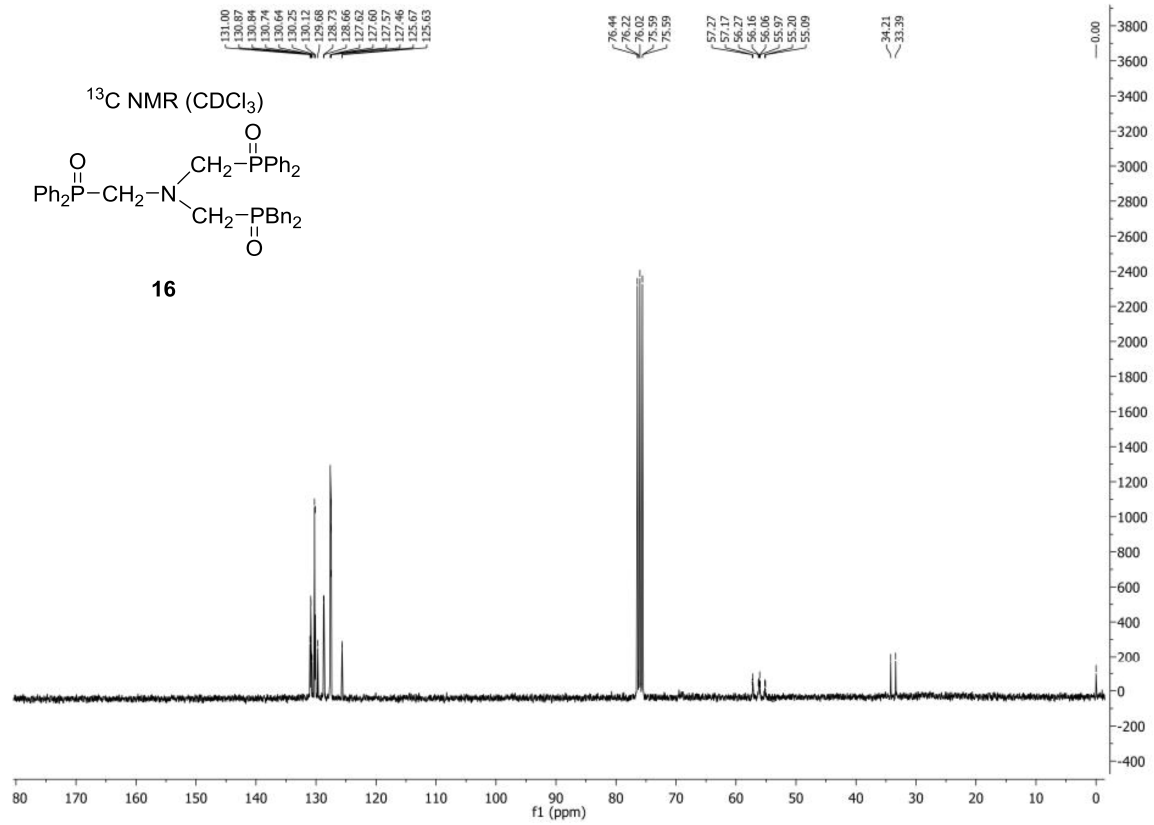

16

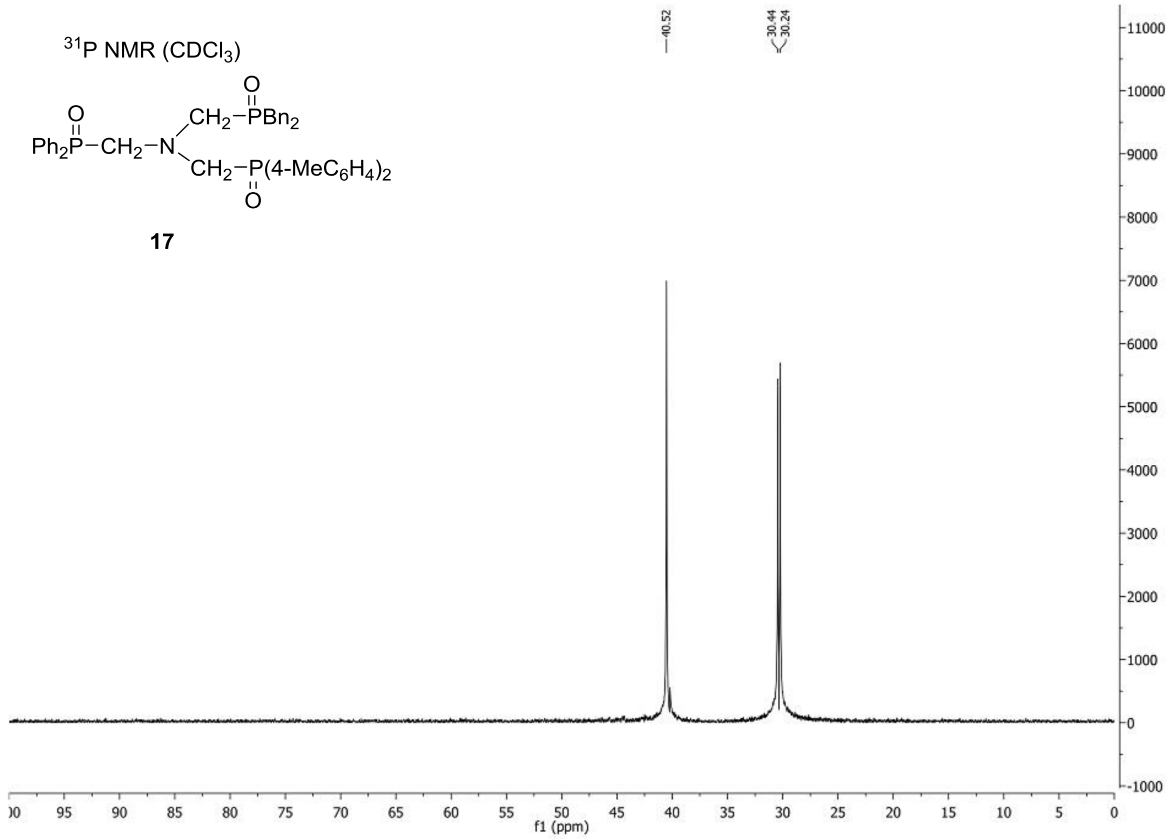

31P NMR (CDCl3)
Ph2P(O)−CH2−N(CH2−P(O)Bn2)(CH2−P(O)(4-MeC6H4)2)
17
40.52
30.44
30.24
f1 (ppm)

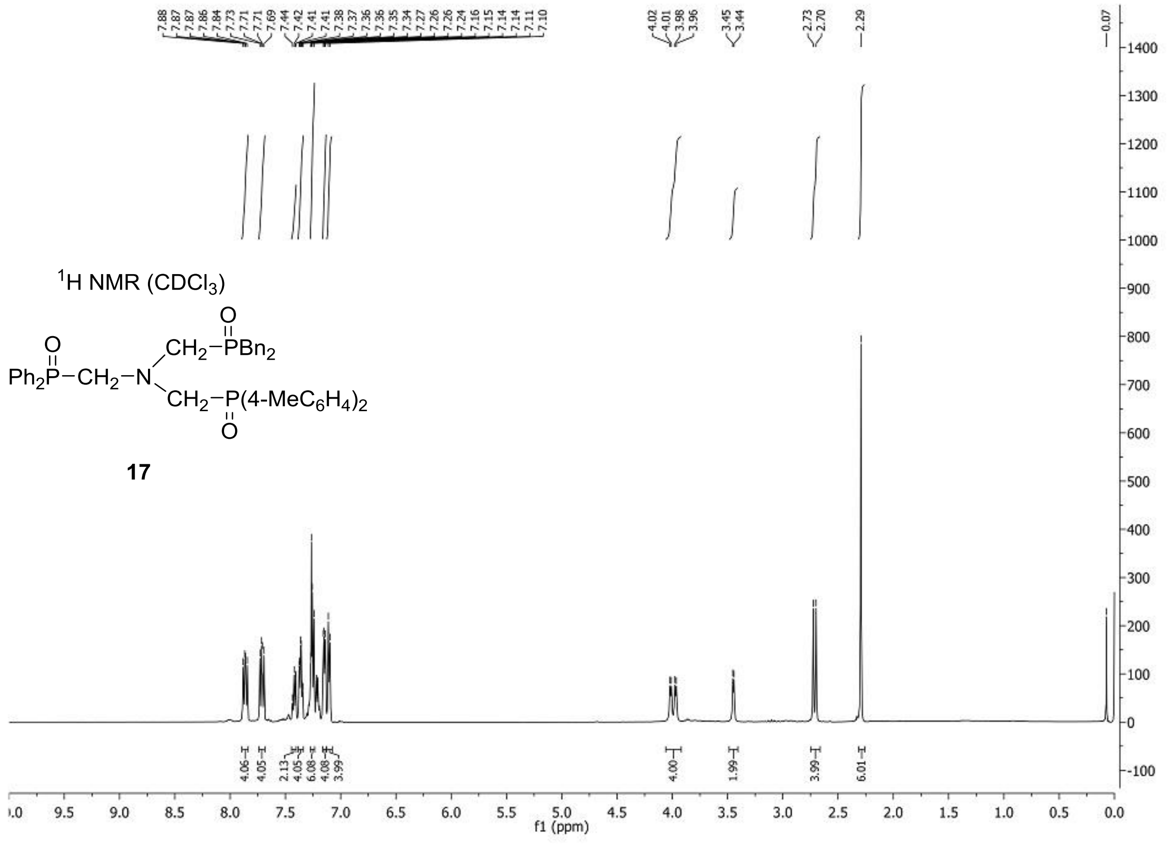

1H NMR (CDCl3)
Ph2P(O)−CH2−N(CH2−P(O)Bn2)(CH2−P(O)(4-MeC6H4)2)
17
4.02
4.01
3.98
3.96
3.45
3.44
2.73
2.70
2.29
0.07
4.00
1.99
3.99
6.01
f1 (ppm)

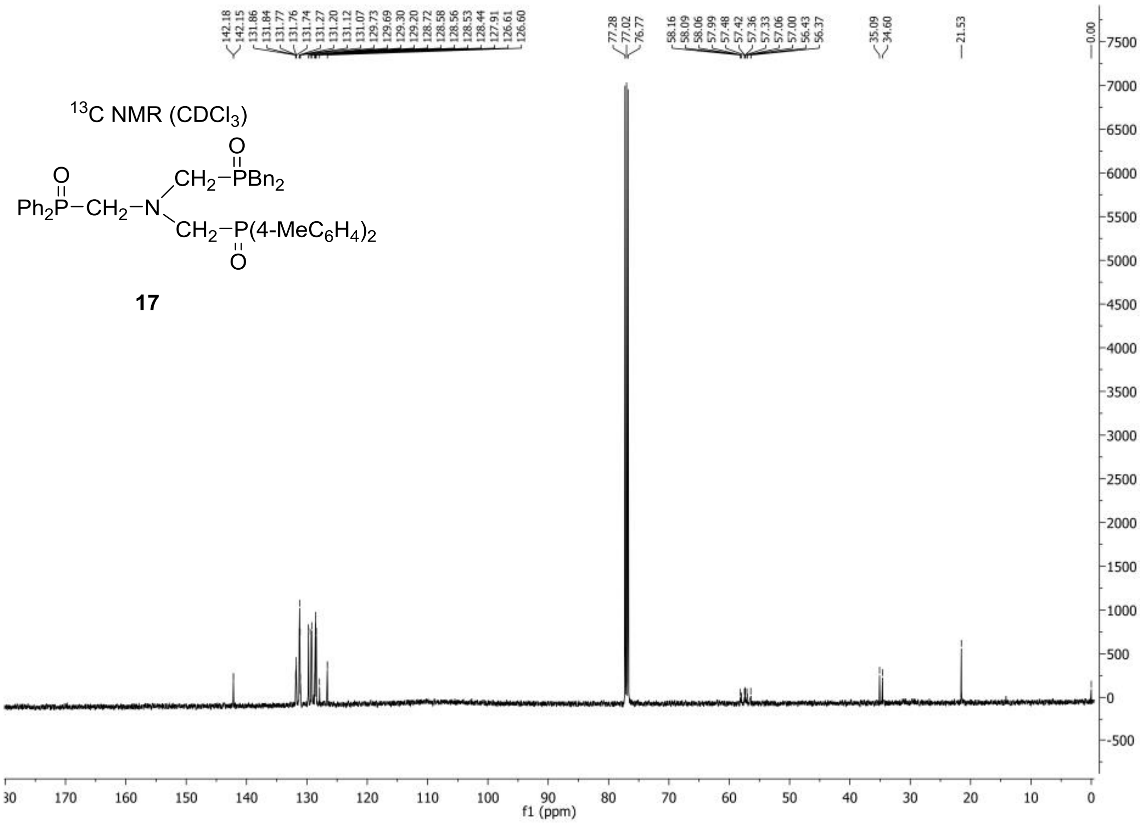
13C NMR (CDCl3)
Ph2P(O)−CH2−N(CH2−P(O)Bn2)(CH2−P(O)(4-MeC6H4)2)
17
142.18 142.15 131.86 131.84 131.77 131.76 131.74 131.27 131.20 131.12 131.07 129.73 129.69 129.30 129.20 128.72 128.58 128.56 128.53 128.44 127.91 126.61 126.60
77.28 77.02 76.77
58.16 58.09 58.06 57.99 57.48 57.42 57.36 57.33 57.06 57.00 56.43 56.37
35.09 34.60
21.53
0.00
f1 (ppm)

References

S1 Bálint, E.; Tripolszky, A.; Jablonkai, E.; Karaghiosoff, K.; Czugler, M.; Mucsi, Z.; Kollár, L.; Pongrácz, P.; Keglevich, G. *J. Organomet. Chem.* **2016**, *801*, 111–121. doi:10.1016/j.jorganchem.2015.10.029

S2 Keglevich, G., Szekrényi, A. *Lett. Org. Chem.* **2008**, *5*, 616–622. doi:10.2174/157017808786857598

S3 Sowa, S., Stankevic, M., Flis, A., Pietrusiewicz,. M. *Synthesis* **2018**, *50*, 2106–2118. doi:10.1055/s-0036-1591546
